# Spatial Distribution of Ammonia Concentrations and Modeled Dry Deposition in an Intensive Dairy Production Region

**DOI:** 10.3390/atmos15010015

**Published:** 2023-12-22

**Authors:** April B. Leytem, John T. Walker, Zhiyong Wu, Kossi Nouwakpo, Colleen Baublitz, Jesse Bash, Gregory Beachley

**Affiliations:** 1 Northwest Irrigation and Soils Research Laboratory, United States Department of Agriculture—Agricultural Research Service, Kimberly, ID 83341, USA; 2 Office of Research and Development, United States Environmental Protection Agency, Durham, NC 27711, USA; 3 RTI International, Durham, NC 27711, USA; 4 Office of Atmospheric Protection, United States Environmental Protection Agency, Washington, DC 20004, USA

**Keywords:** ammonia, dairy, dry deposition, bidirectional exchange

## Abstract

Agriculture generates ~83% of total US ammonia (NH_3_) emissions, potentially adversely impacting sensitive ecosystems through wet and dry deposition. Regions with intense livestock production, such as the dairy region of south-central Idaho, generate hotspots of NH_3_ emissions. Our objective was to measure the spatial and temporal variability of NH_3_ across this region and estimate its dry deposition. Ambient NH_3_ was measured using diffusive passive samplers at 8 sites in two transects across the region from 2018–2020. NH_3_ fluxes were estimated using the Surface Tiled Aerosol and Gaseous Exchange (STAGE) model. Peak NH_3_ concentrations were 4–5 times greater at a high-density dairy site compared to mixed agriculture/dairy or agricultural sites, and 26 times greater than non-agricultural sites with prominent seasonal trends driven by temperature. Annual estimated dry deposition rates in areas of intensive dairy production can approach 45 kg N ha^−1^ y^−1^, compared to <1 kg N ha^−1^ y^−1^ in natural landscapes. Our results suggest that the natural sagebrush steppe landscapes interspersed within and surrounding agricultural areas in southern Idaho receive NH_3_ dry deposition rates within and above the range of nitrogen critical loads for North American deserts. Finally, our results highlight a need for improved understanding of the role of soil processes in NH_3_ dry deposition to arid and sparsely vegetated natural ecosystems across the western US.

## 1. Introduction

In the U.S., 5.5 million tons of ammonia (NH_3_) are generated each year, with ~83% of that originating from agriculture [1]. Approximately 60% of U.S. agricultural emissions are generated from livestock production. Ammonia can adversely impact sensitive ecosystems through dry deposition as NH_3_ and wet deposition following conversion to ammonium (NH_4_), creating eutrophication, decline of biodiversity and soil acidification [2–4]. Across the U.S., dry deposition contributes approximately 55% to total deposition and is the dominant process over broad areas of the southwest and other arid regions of the west [5]. The spatial variation of atmospheric NH_3_ concentration is related to local sources, with livestock production generating hotspots of NH_3_ emissions and concentrations in the landscape. As NH_3_ has an atmospheric lifetime on the order of hours, a large proportion of emitted NH_3_ can be deposited within a few kilometers of the source [2,6,7].

Mapping of NH_3_ concentrations in the U.S. utilizing satellite retrievals indicates that the highest concentrations are in the Central Valley of California, the Snake River valley in Idaho and the western High Plains [8]. Idaho is one of the top milk-producing states in the U.S, ranking third in 2020 [9]. Idaho’s dairy cow inventory has increased by 73% over the last two decades, consisting of 652,000 head of lactating cattle in 2021 [10], ~75% of which are located in the Snake River valley in an area known as the Magic Valley. Leytem et al. [11] estimated that approximately 31,000 MT of NH_3_-N was generated from dairy production in the Magic Valley and another 13,000 MT of NH_3_-N was generated from land application of fertilizer. The transport and fate of agricultural NH_3_ emissions in the Magic Valley is of interest as there are ecosystems within and downwind of the region that are sensitive to nitrogen deposition, including native rangeland and high-altitude national forests and parks [12].

While regional patterns of wet deposition (NH_4_^+^) are relatively well known from long-term monitoring by the National Atmospheric Deposition Program/National Trends Network (NADP/NTN), patterns of dry deposition (NH_3_) are less understood due to a lack of routine direct flux measurements. While short-term intensive flux studies have been conducted in some grassland and forest ecosystems, primarily in the eastern and midwestern U.S., measurements in arid western landscapes remain lacking [13]. NH_3_ may be emitted from, or deposited to, soil and vegetation depending on the difference between the surface compensation point and atmospheric concentration, so that NH_3_ dry deposition is generally represented using the typical resistance analogy in a bidirectional flux framework [14–17]. Estimates of NH_3_ dry deposition for regional-scale critical loads and other ecosystem exposure assessments are typically derived from gridded chemical transport models (CTMs) [18–21]. In CTMs, accurate prediction of NH_3_ dry deposition rates is dependent on accurate simulation of atmospheric concentrations and, therefore, emissions, dispersion and transport, and atmospheric chemical transformations. Ammonia dry deposition rates may also be estimated using in-situ inferential (i.e., field-scale) modeling [22,23], which utilizes measurements of air-concentration, meteorology, and surface characteristics to provide site-specific deposition rates. In the absence of long-term measurements of the flux itself, inferential modeling using in-situ measurements yields a more constrained estimate of NH_3_ bidirectional exchange than a CTM using the same flux algorithms.

The first objective of this study was to assess the spatial and temporal variability of ambient atmospheric NH_3_ concentrations across the Magic Valley region of Idaho. A second objective was to use the measured air concentrations, along with measurements of meteorology, biogeochemistry, and other surface characteristics to model dry deposition of NH_3_ across the region. The bidirectional modeling framework was used to characterize seasonal and annual patterns of fluxes across sites and their relationships to soil and vegetation exchange pathways. Differences between in-situ dry deposition estimates and those from a commonly used CTM were evaluated. Finally, model sensitivity testing was used to identify additional measurements needed to better understand NH_3_ flux processes in similar Western ecosystems.

## 2. Materials and Methods

### 2.1. Study and Site Descriptions

**Figure 1.**
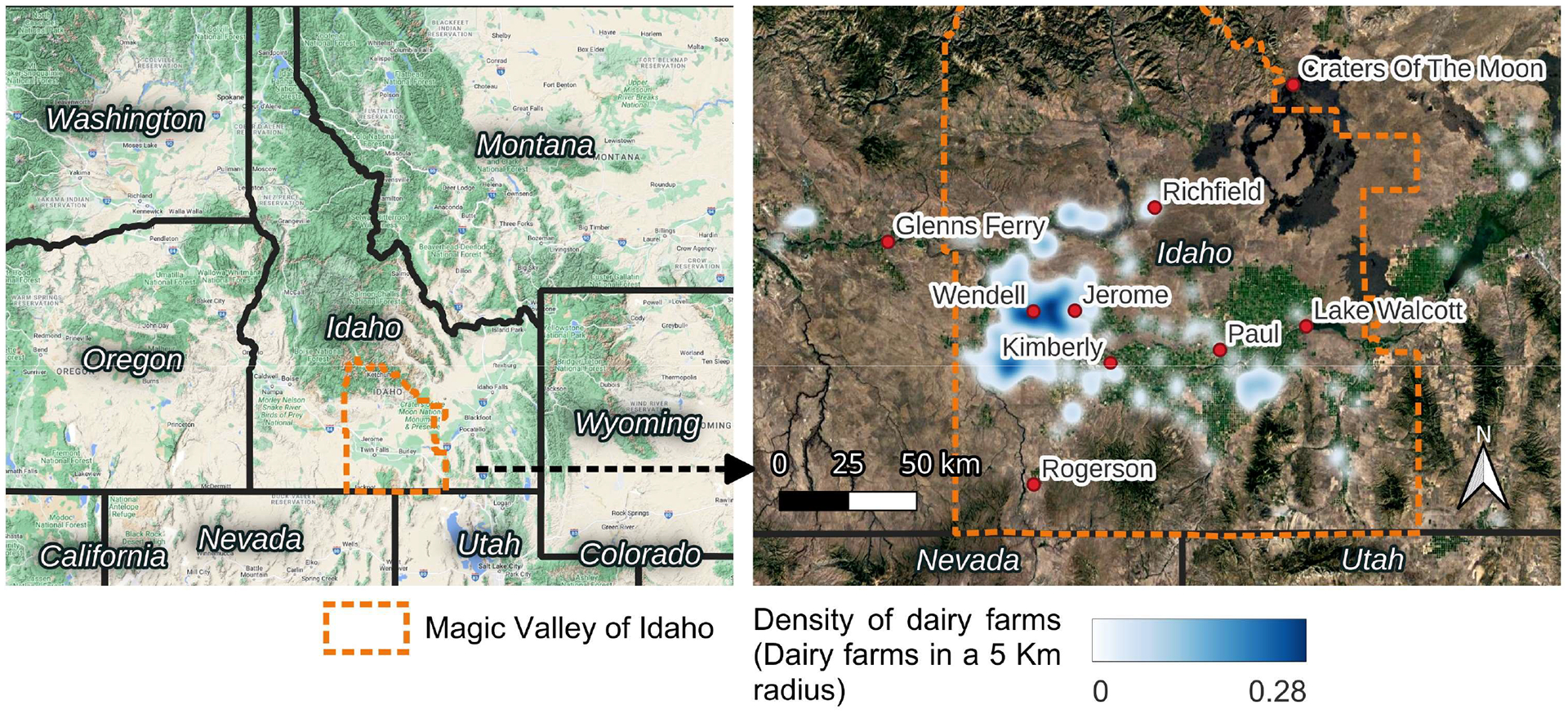
Study location and sites along with the spatial distribution of dairy farms within the region and delineation of the Magic Valley in south-central Idaho.

Ambient atmospheric NH_3_ was measured using diffusive passive samplers (Radiello) in two transects (north–south and west–east) across the Magic Valley region of south-central Idaho (Figure 1) with 6 sampling locations monitored biweekly from February 2018 to December 2020, a 7th site monitored biweekly from February 2018 to March 2020, and an 8th site monitored biweekly from December 2019 to December 2020. The locations were chosen to reflect a gradient of agricultural/dairy production intensity across the region based on dairy and cropland activity (Table 1) and to account for wind direction, which is predominantly from the west. Figure 1 shows the density of dairy operations across the Magic Valley region, with the highest concentrations being in the western half of the region in Wendell (intensive dairy) and Jerome, ID (agriculture/dairy).

The west–east transect started at Glenns Ferry (GF, agriculture), which lies along the Snake River west of the Magic Valley, and is a mix of irrigated agriculture and sagebrush steppe with no dairy farms. Land use is >50% sagebrush steppe within 5 km of the sampling location. The next location to the southeast was in Wendell (WE), which is the most densely populated dairy region in the Magic Valley. The closest dairy was 1.4 km to the north, and there were ~20 dairies within a 5 km radius of the sampling location. The sampling site in Jerome (JE) is due east of WE. This site was added later in this study to capture the gradient between WE, which had the highest NH_3_ concentrations, and Kimberly (KI, agriculture/dairy), which had an intermediate concentration. The sampler was located in a low-density urban area, and there were approximately 14 dairies within 5 km, and the closest dairy was 1.8 km north of the sampling location. Southeast of JE was the sampling site in KI, which is mainly an agricultural area with 1 dairy, low-density rural development, and a small urban area within 5 km. To the east of KI was the sampling site in Paul (PA, agriculture/dairy), which is surrounded by agricultural fields, 3 dairies, and 1 sheep feedlot within 5 km. The farthest east site was Lake Walcott (LW, agriculture), which had some agricultural fields to the west, with the remaining area within 5 km (>50% of the land area) being sagebrush steppe or the lake itself. The sampler was located within the Minidoka National Wildlife Refuge and represented the eastern downwind edge of the Magic Valley region.

The north–south transect started at Craters of the Moon National Monument (COTM, minimal agriculture) which is approximately 65 km to the north of the northern edge of the Magic Valley. The area within 15 km of the site is sagebrush steppe and lava fields. This site represents an area with minimal agricultural impact. To the southwest of COTM was the Richfield (RI, agriculture) site, which is ~50% agriculture and 50% sagebrush steppe within 5 km of the sampler location, with 1 dairy 4 km to the SW and one small beef facility 1.2 km to the west. The KI site was used as the central point in the Magic Valley along the transect. To the southwest of KI was the Rogerson (RO, minimal agriculture) site, which was approximately 30 km south of the Magic Valley, located in a predominantly sagebrush steppe area, which would also represent an area with minimal agricultural impact. The wind at this location is predominantly from the south.

The sampler locations were chosen to be free of obstacles that could impede wind flows, except for the JE site which was located in an urban area. Locations also had to be at least 1 km from nearby dairies to avoid point-source NH_3_ emissions. At the start of this study, the KI site was established as an official National Atmospheric Deposition Program (NADP) Ammonia Monitoring Network (AMoN) site, and we deployed additional samplers at that location that were analyzed in-house to compare to the data generated by the NADP AMoN network. Ammonia concentrations from the NADP AMoN site located at COTM were utilized in this study. Additional information on AMoN and national network data can be found at: https://nadp.slh.wisc.edu/networks/ammonia-monitoring-network/ (Accessed on 12 December 2023).

### 2.2. Sampler Preparation, Analyses and Laboratory Comparison

The NADP AMoN network protocols were followed to provide data consistent with the AMoN network database. As mentioned, ambient NH_3_ concentrations were measured with diffusive NH_3_ samplers (Radiello). These were deployed biweekly in duplicate, providing 2-week mean surface NH_3_ concentrations. Samplers were prepared in the laboratory and transported to and from the sites in sealed containers. Samplers were set at a height of 2 m aboveground and mounted on steel posts, with inverted plastic buckets used to protect the sampler from rain and direct sunlight. During each deployment, a trip blank was carried to the field sites in a sealed container to assess potential contamination during transport and was handled and processed the same way the exposed samplers were.

The Radiello samplers consist of a cylindrical diffusive body (60 mm height × 16 mm diameter) in which a chemiadsorbent cartridge (60 mm long × 5.8 mm in diameter) impregnated with phosphoric acid is inserted. Ammonia is absorbed in the form of ammonium ion (NH_4_^+^). Prior to assembly, bodies were cleaned by sonicating in DI water with heat for 4 h (sonicate 3 h drain and rinse bodies, then sonicate 1 h), soaking overnight, then sonicating 4 h (sonicate 2 h, drain and rinse bodies, then sonicate 2 h) the next day, then allowed to dry in a clean hood. Sampler components were assembled in a clean hood immediately prior to deployment. Exposed samplers were refrigerated (24 h max) at 4 °C prior to extraction and colorimetric analyses. Samplers were disassembled in a clean hood, and the core was placed in a 15 mL vial containing 10 mL of DI water, sonicated for 20 min, and then soaked for 24 h in the refrigerator; then, cores were removed, and samples were vortexed for 30 s, and extractant was transferred and analyzed on the Lachat QuikChem 8500 Flow Injection Analysis System (Hach, Loveland, CO, USA) using the Lachat QuikChem Method 10-107-06-1-J for determination of NH_4_.

The NH_4_^+^ concentration of extract solution for each site was determined by subtracting the concentration of NH_4_^+^ in the trip blank from the exposed samplers at each location (Equation (1)).

(1) $$C_{\text{NH}4,\;\text{Ex}\cdot }=C_{e}-C_{b}$$

where C_NH4_, _Ex._ is the NH_4_^+^ concentration of extract solution (mg L^−1^), C_e_ is the NH_4_^+^ concentration of the exposed sampler (mg L^−1^) and C_b_ is the NH_4_^+^ concentration of the trip blank (mg L^−1^). The NADP protocol does not adjust for trip blanks, however, due to the high ambient NH_3_ concentrations in our region we did correct for the trip blanks. The subtraction of the blanks reduced concentrations by an average of 4.8% (91% of samples were reduced by <10%). See below for a discussion of the comparison of in-house sample analysis compared to the NADP laboratory.

The ambient concentration of atmospheric NH_3_ was then calculated with the following equation:

(2) $$C_{NH3},\mu \text{g}/m^{3}=\left(0.94412\times C_{NH4,\;Ex.}\times V_{Ex}.\times 10^{6}\right)÷\left(\beta_{NH3}\times Q_{NH3NH3}\times t\right)$$

where, C_NH3_, μg/m^3^ is the ambient NH_3_ gas concentration in micrograms per cubic meter of air, 0.94412 is the molecular weight ratio converting measured NH_4_^+^ ion to NH_3_ gas, 10^6^ represents unit conversion, V_Ex._ is the extract solution volume (mL), β_NH3_ is the passive sampler empirical mass transfer correction factor based on NH_3_ monitoring field data (1.19, unitless), Q_NH3_ is the passive sampler flow rate (198, mL per min), and t is the sampler exposure time (min). These calculations assume atmospheric temperature and pressure conditions of 20 °C and 1 atm. As stated by the manufacturer, Q_NH3_ is negligibly effected by atmospheric pressure, humidity in the range of 10–90%, wind speed between 0.1 and 10 m s^−1^, and temperature in the range from 2–39 °C [24]. Average conditions over the sampling periods were within the stated ranges for humidity and wind speed. While average temperatures never exceeded the upper threshold, there were some instances where temperatures were lower (<1 to 3%) than 2 °C, however as the NADP protocol does not adjust for temperature, to be consistent, we did not correct for this.

Over the study period, there were 60 measurements made at the KI site with ambient NH_3_ concentrations determined by both the NADP laboratory and the in-house laboratory at Kimberly, ID. There was good agreement in results from the two laboratories (r^2^ = 0.995) with the in-house laboratory being biased slightly high by 5%. The greatest deviations were at ambient NH_3_ concentrations above 14 μg m^−3^.

### 2.3. Modeling

A newly developed bidirectional air-surface exchange scheme used in the Community Multi-scale Air Quality Model (CMAQ) version 5.3 [17], the Surface Tiled Aerosol and Gaseous Exchange (STAGE) model, is used in this study to estimate the NH_3_ fluxes. Previously, STAGE was evaluated for ozone as a participating model in the Air Quality Model Evaluation International Initiative 4 [25], and for reactive nitrogen deposition against measurements from a deciduous forest in the Appalachian Mountains [26].

### 2.4. Description of the STAGE Model

The STAGE model parameterizes the air-surface exchange of gases as a gradient process following the widely used resistance model of Nemitz et al. [14] and Massad et al. [27]:

(3) $$F=-f_{\text{veg}}\frac{\chi_{a}(z)-\chi_{z_{0}}}{R_{a}}-\left(1-f_{\text{veg}}\right)\frac{\chi_{a}(z)-\chi_{g}}{R_{a}+R_{g}}$$

where *F* is the net flux above the canopy (a negative value represents a net deposition flux and a positive value represents a net emission flux); $\chi_{a}(z)$ is the ambient concentration at a reference height $(z);\chi_{z0}$ is the compensation point at height *d* (displacement height) + *z*_0_ (roughness length); *χ*_*g*_ is the ground layer compensation point; *R*_*a*_ is the aerodynamic resistance between *z* and *d* + *z*_0_; *R*_*g*_ is the total ground resistance including in-canopy aerodynamic resistance (*R*_*inc*_), ground boundary layer resistance (*R*_*bg*_), and soil resistance (*R*_*soil*_) (*R*_*g*_ = *R*_*inc*_ + *R*_*bg*_ + *R*_*soil*_); and *f*_*veg*_ is the vegetation coverage fraction. The compensation point *χ*_*z*0_ is estimated following Nemitz, et al. [14] as:

(4) $$\chi_{z0}=\frac{\frac{\chi_{a}}{R_{a}}+\frac{\chi_{l}}{R_{b}}+\frac{\chi_{g}}{R_{g}}}{{\left(R_{a}\right)}^{-1}+{\left(R_{b}\right)}^{-1}+{\left(R_{g}\right)}^{-1}}$$

where *R*_*b*_ is the quasi-laminar boundary layer at the leaf/vegetation surface [27]. The compensation point above the leaf (*χ*_*l*_) is estimated following Nemitz et al. [14] with the addition of a cuticular compensation point:

(5) $$\chi_{l}=\left(\chi_{a}{\left(R_{a}R_{b}\right)}^{-1}+\chi_{s}\left({\left(R_{a}R_{s}\right)}^{-1}+{\left(R_{b}R_{s}\right)}^{-1}+{\left(R_{g}R_{s}\right)}^{-1}\right)+\chi_{\text{cut}}\left({\left(R_{a}R_{\text{cut}}\right)}^{-1}+{\left(R_{b}R_{\text{cut}}\right)}^{-1}+{\left(R_{g}R_{\text{cut}}\right)}^{-1}\right)+\chi_{g}{\left(R_{b}R_{g}\right)}^{-1}\right)÷\left[{\left(R_{a}R_{b}\right)}^{-1}+{\left(R_{a}R_{s}\right)}^{-1}+{\left(R_{a}R_{\text{cut}}\right)}^{-1}+{\left(R_{b}R_{s}\right)}^{-1}+{\left(R_{b}R_{\text{cut}}\right)}^{-1}+{\left(R_{b}R_{g}\right)}^{-1}+{\left(R_{g}R_{s}\right)}^{-1}+{\left(R_{g}R_{\text{cut}}\right)}^{-1}\right]$$

where *R*_*s*_ and *R*_*cut*_ are the stomatal and cuticular resistances, respectively, and *χ*_*s*_, *χ*_*cut*_, and *χ*_*g*_ are the stomatal, cuticular, and ground compensation points, respectively.

At a vegetated site, the component fluxes are calculated as:

(6) $$F_{s}=-\frac{\chi_{l}-\chi_{s}}{R_{s}}$$

(7) $$F_{\text{cut}}=-\frac{\chi_{l}-\chi_{\text{cut}}}{R_{\text{cut}}}$$

(8) $$F_{g}=-\frac{\chi_{z_{0}}-\chi_{g}}{R_{g}}$$

where *F*_*s*_, *F*_*cut,*_ and *F*_*g*_ are the fluxes to leaf stomata, leaf cuticle, and ground surface, respectively. The net flux over canopy is the sum of the component fluxes (*F*_*net*_ = *F*_*s*_ + *F*_*cut*_ + *F*_*g*_).

The stomatal, cuticular, and ground compensation points (*χ*_*s*_, *χ*_*cut*_, *χ*_*g*_) are described according to Nemitz et al. [14] as a function of temperature (*T*) and the emission potentials (Γ):

(9) $$\chi_{s,cut,g}=\frac{161512}{T}10^{\frac{-4507.11}{T}}\Gamma_{s,cut,g}$$

Γ_cut_ is set to 0 in this study and thus there is only deposition to leaf cuticles, which is consistent with typical implementation of current bidirectional exchange models. In the case of NH_3_, the foliage and ground layers may act as a source or sink of NH_3_ depending on the ratio of the ambient concentration to the respective compartment compensation point [28]. Here values of Γ for NH_3_ are derived from measurements of live vegetation and soil chemistry as described below. Values used in the base model simulation are listed in Table 2 and further described below in Section 2.9.

### 2.5. Model Configuration

The STAGE model is extracted from the CMAQ v5.3 and executed in a 1-D (standalone) mode. The standalone in-situ model requires inputs of hourly NH_3_ concentration, hourly meteorological forcing, and site-specified biogeochemical parameters including land use type, leaf area index, and NH_3_ emission potentials of vegetation and soil. The meteorological inputs include precipitation rate (*P*_*recip*_), relative humidity (*RH*), air temperature (*T*_*a*_), surface wetness (*SW*), atmospheric pressure (*P*_*a*_), soil moisture (*SM*), soil temperature (*T*_*soil*_), wind speed (*WS*), friction velocity (u_*_), and downward shortwave radiation (*R*_*g_in*_).

### 2.6. Hourly NH_3_ Concentrations

Air concentrations measured with the passive samplers are integrated over a two-week period. However, the NH_3_ flux will be influenced by diurnal variability in the air concentration, which may display different patterns across sites depending on proximity to sources, complexity of terrain, and meteorology. Hourly measurements sufficient to assess diurnal variability across study sites and over time are not available for our study area. Alternatively, we have incorporated diurnal patterns by temporally scaling the two-week integrated measured concentration to the hourly time-step using output from CMAQ V5.2.3 developed in a separate study [29]. Hourly NH_3_ concentrations for in-situ STAGE simulations were generated by combining the in-situ bi-weekly measurements and hourly CMAQ concentrations according to:

(10) $${\left[NH_{3}\right]}_{\left(i,j\right)}={\left[NH_{3}\right]}_{\left(i,j\right)}^{\text{CMAQ}}\times \frac{{\left[\bar{NH_{3}}\right]}_{\left(w,j\right)}^{obs}}{{\left[\bar{NH_{3}}\right]}_{\left(w,j\right)}^{\text{CMAQ}}}$$

where ${\left[{NH}_{3}\right]}_{\left(i,j\right)}$ represents the STAGE NH_3_ concentration at the *i*th hour of the study period for the *j*th site, ${\left[{NH}_{3}\right]}_{\left(i,j\right)}^{\text{CMAQ}}$ is the corresponding CMAQ-modeled concentration at the *i*th hour of the study period for the *j*th site, and $\frac{{\left[\bar{NH_{3}}\right]}_{\left(\text{w},j\right)}^{\text{obs}}\;}{{\left[\bar{{NH}_{3}}\right]}_{\left(w,j\right)}^{\text{CMAQ}}}$ is a scaling factor, which is a ratio of the biweekly mean NH_3_ concentration between the in-situ measurements and the CMAQ model. *w* indicates the *w*th biweek, which covers the *i*th hourly data point. This was done for each study site using CMAQ output for the corresponding 12 km grid cell. This approach introduces diurnal variability of the air concentration represented in CMAQ but does not incorporate average bias of the CMAQ predicted air concentration. That is, the two-week average of the hourly scaled concentration used in the in-situ STAGE simulation is exactly equal to the corresponding measured two-week integrated concentration.

### 2.7. Meteorology

The hourly meteorological data were derived from station measurements and various model outputs. The station measurements are from the cooperative agricultural weather network (AgriMet) and the data can be accessed from https://www.usbr.gov/pn/agrimet/wxdata.html (Accessed on 15 December 2023). For each study site, the measured meteorological data were used first if there was a meteorology station available within a distance of 20 km and the data were not missing. Otherwise, Real-Time Mesoscale Analysis (RTMA) products were extracted to provide the hourly data for air temperature, relative humidity, wind speed, and atmospheric pressure and the North American Land Data Assimilation System (NLDAS) products were used for downward shortwave radiation and precipitation rate. RTMA is a high-spatial and temporal resolution analysis for near-surface weather conditions, which includes hourly analyses at 2.5 km for the continental US. NLDAS construct quality-controlled, and spatially and temporally consistent, land-surface model (LSM) datasets on a 0.125° (~12 km) grid from the best available observations and reanalysis. More information about the meteorology products can be found at https://www.nco.ncep.noaa.gov/pmb/products/rtma/ (Accessed on 15 December 2023) and https://ldas.gsfc.nasa.gov/nldas (Accessed on 15 December 2023).

Besides the standard meteorological variables, STAGE requires inputs of friction velocity, surface wetness, soil moisture, and soil temperature, which are not available from the station measurements or the RTMA/NLDAS products. The Noah LSM [30] was used to generate hourly data for these variables, executed in a 1-D mode, and driven by the integrated meteorological datasets from the station measurements and the RTMA/NLDAS products. Friction velocities were calculated in Noah via an iterative process using temperature, relative humidity, wind speed, and air pressure [31]. Noah calculates the canopy surface moisture budget [30], which is used to derive the surface wetness condition. A multi-level soil sub-model is implemented in Noah in which soil temperature and moisture were calculated at the depths of 0.1 m, 0.3 m, 0.6 m and 1.0 m. The sources of meteorological data for each site are listed in Table 3.

### 2.8. Land Use and Leaf Area Index

The MODerate resolution Imaging Spectroradiometer (MODIS) land cover type product (MCD12Q1, Version 5.1), which provides global land cover types at yearly intervals with 500 m pixel size, was used to obtain the land use and land cover information for each site. The MODIS land cover data from 2018 to 2019 were used to generate average land cover fractions within a 1 km radius surrounding each site (see Table 2).

**Figure 2.**
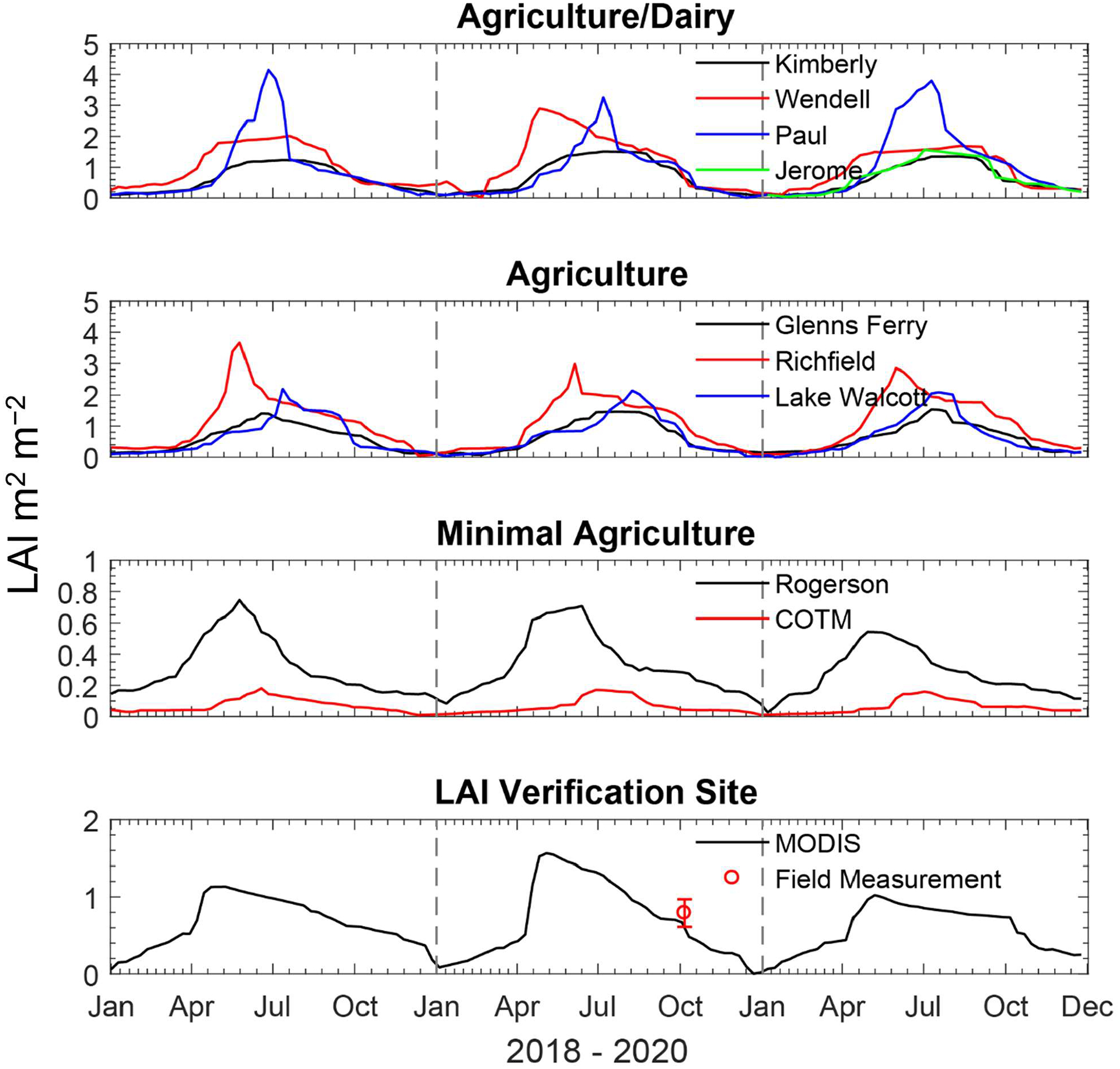
MODIS leaf area index at each site. The lines are data extracted from the MODIS products. The open circle and error bar in the bottom panel shows the mean and standard deviation of field LAI measurements.

A continuous time series of leaf area index (LAI) was extracted from the MODIS global LAI product (MCD15A2H, version 6) which is an 8-day composite dataset with 500 m pixel size. The LAI data within a 1 km radius surrounding each site were extracted to generate average LAI values. The raw MODIS data were filtered using the MODIS quality control (QC) layers, including CloudState, Confidence Score, Snow_Ice, Aerosol, Cirrus, Internal_CloudMask, and Cloud_Shadow flags then averaged, smoothed and gap filled. The LAI values that were extracted for each site are shown in Figure 2 along with a ground-based measurement (LAI-2200C plant canopy analyzer, LI-COR, Inc., Lincoln, NE, USA) conducted during the fall of 2019 at a research site near Kimberly, ID, characterized by a mixture of grass and sagebrush.

The open circle and error bar in the bottom panel shows the mean and standard deviation of field LAI measurements.

### 2.9. Vegetation and Soil Emission Potentials

Vegetation emission potentials (Γ_s_) were estimated from measurements of NH_4_^+^ concentration and pH in ground up tissue using the headspace equilibration procedure described by Walker et al. [26]. Crops are assigned a value of 4750 derived from seasonal measurements of corn and soybean over the course of a year in Bondville, IL [32]. Grasslands (Γ_s_ = 1600) reflect a median value from measurements across multiple sites (Chapel Hill, NC, USA; Bondville, IL, USA; Chiricahua, NM, USA)[32], including new measurements from Idaho. In model simulations, the “grassland” land use category reflects a 50/50 combination of Γ_s_ for grassland and sagebrush (Γ_s_ = 685). A weighted average value is determined from the land use fraction (Table 2) for sites designated as a mix of croplands and grassland.

In the STAGE model, the ground emission potential (Γ_g_ = [NH_4_^+^]/[H^+^]) is calculated from the concentration of NH_4_^+^ in the soil solution (S_NH4+_) estimated using a sorption model [33,34]. The NH_4_^+^ concentration is then combined with measured soil pH to estimate Γ_g_. The sorption modeling approach partitions the measured total extractable NH_4_^+^ between the soil matrix, which is unavailable for air-surface exchange, and the soil pore water. In this case, a Langmuir model [35–37] is used to simulate the soil sorption characteristics using the maximum sorption capacity (Q_max_, mg NH_4_^+^ kg^−1^ soil) and binding coefficient (K_L_, L mg^−1^) determined via a sorption curve.

The sorption curve is developed by adding 3 g of soil to 30 mL of solution containing NH_4_^+^ at initial concentrations (C_0_) of 0, 5, 10, 25, 50, 75, 100, 200, 300, and 500 mg NH_4_^+^ L^−1^ (as NH_4_Cl). After equilibrating for 24 h, the concentration of NH_4_^+^ in solution (C_e_) is determined. The amount of NH_4_^+^ sorbed to the soil matrix (Sr_NH4+_) is calculated from the difference between C_e_ and C_0_, accounting for the baseline amount of total extractable NH_4_^+^ in the soil (T_NH4+_) determined by 1M KCl extraction. Sorption parameters are determined via the Langmuir model

(11) $$Sr_{NH4+}=\frac{ASl_{NH4+}}{1+BSl_{NH4+}}$$

where A = Q_max_K, B = K = 0.0084 L mg^−1^ and Q_max_ = A/K = 1473 mg NH_4_^+^ kg^−1^ soil. The mass balance for soil total extractable NH_4_^+^ (T_NH4+_, mg kg^−1^) is:

(12) $$T_{NH4+}=Sr_{NH4+}+\theta Sl_{NH4+}+\theta Sl_{NH3}$$

where Sr_NH4+_ is the amount of NH_4_^+^ sorbed to the soil, Sl_NH4+_ is the concentration of NH_4_^+^ in the soil solution, Sl_NH3_ is the concentration of NH_3_ in the soil solution, and θ is soil mass wetness (L H_2_O kg^−1^ soil). At soil pH < 8.0, Sl_NH3_ will contribute < 5% of Sl_NH4+_ + Sl_NH3_ and has thus been ignored in this case for simplicity. As noted by Venterea et al. [34], assumptions regarding whether Sl_NH3_ is captured in the T_NH4+_ analysis therefore makes a small difference. With this simplification, Equation (11) is substituted into Equation (12) yielding:

(13) $$T_{NH4+}=\frac{Q_{m}KSl_{NH4+}}{1+KSl_{NH4+}}+\theta Sl_{NH4+}$$

After the Langmuir constants (K and Q_m_) have been determined, Sl_NH4+_ is calculated from measurements of T_NH4+_ and θ using the quadratic formula as:

(14) $$\text{X}=\frac{-A-\text{C}+YB+\sqrt{Y^{2}B^{2}-2YAB+2YB\text{C}+A^{2}+{\text{C}}^{2}+2A\text{C}}}{2B\text{C}}$$

where X = Sl_NH4+_, Y = T_NH4+_, A = Q_m_K, B = K, C = θ. The soil emission potential derived from the Langmuir isotherm (Γ_g_) is then calculated as:

(15) $$\Gamma_{g}=\frac{S_{lNH4+}}{10^{-pH}}$$

where Sl_NH4+_ is expressed in units of M (i.e., mol L^−1^). The corresponding soil compensation point derived from the Langmuir isotherm (X_g_) is then calculated via Equation (9).

For STAGE model simulations, Γ_g_ is characterized from measurements of soil T_NH4+_ and pH in cropland and non-agricultural (grassland/sagebrush) land use categories. For non-agricultural land, T_NH4+_ was measured in 5 locations in a sagebrush/grassland ecosystem in 2019 and 2021 near Kimberly, ID, with each sampling location representing several cores composited from within a 1 m × 1 m area on which duplicate 1M KCl extractions were performed (average = 2.45 ± 1.2 mg NH_4_^+^ kg^−1^ soil, N = 22). Combining T_NH4+_ with a pH value of 7.92 derived from measurements on unfertilized control plots in a long-term nutrient cycling study at the USDA Northwest Irrigation and Soils Research Laboratory in Kimberly, ID, a static value of Γ_g_ = 900 was used for “grassland” simulations. For croplands, a value of Γ_g_ = 3000 was derived from average T_NH4+_ (7.37 ± 1.5 mg NH_4_^+^ kg^−1^ soil, 0–15 cm depth) and pH (7.96) measured on long-term fertilized plots in the same study.

In this analysis, extractable NH_4_^+^ measured on the long-term fertilized plots is assumed to reflect postgrowing season residual soil N. The corresponding Γ_g_ (3000) is used as a baseline value that is then scaled using estimates of Γ_g_ simulated by STAGE as implemented in CMAQ [29] (referred to here as STAGE-CMAQ). Using initial conditions from the Environmental Policy Integrated Climate model (EPIC) [8,38,39], STAGE-CMAQ tracks the mass balance of soil NH_4_^+^ after fertilization through the processes of mineralization, nitrification, and NH_3_ loss to the atmosphere [17,40]. Combining soil NH_4_^+^ concentration and pH, STAGE-CMAQ estimates Γ_g_ at the daily timestep for 21 different cropping systems [38,40]. For this analysis, 2018 STAGE-CMAQ simulated Γ_g_ (0–1 cm layer) was analyzed [29].

**Figure 3.**
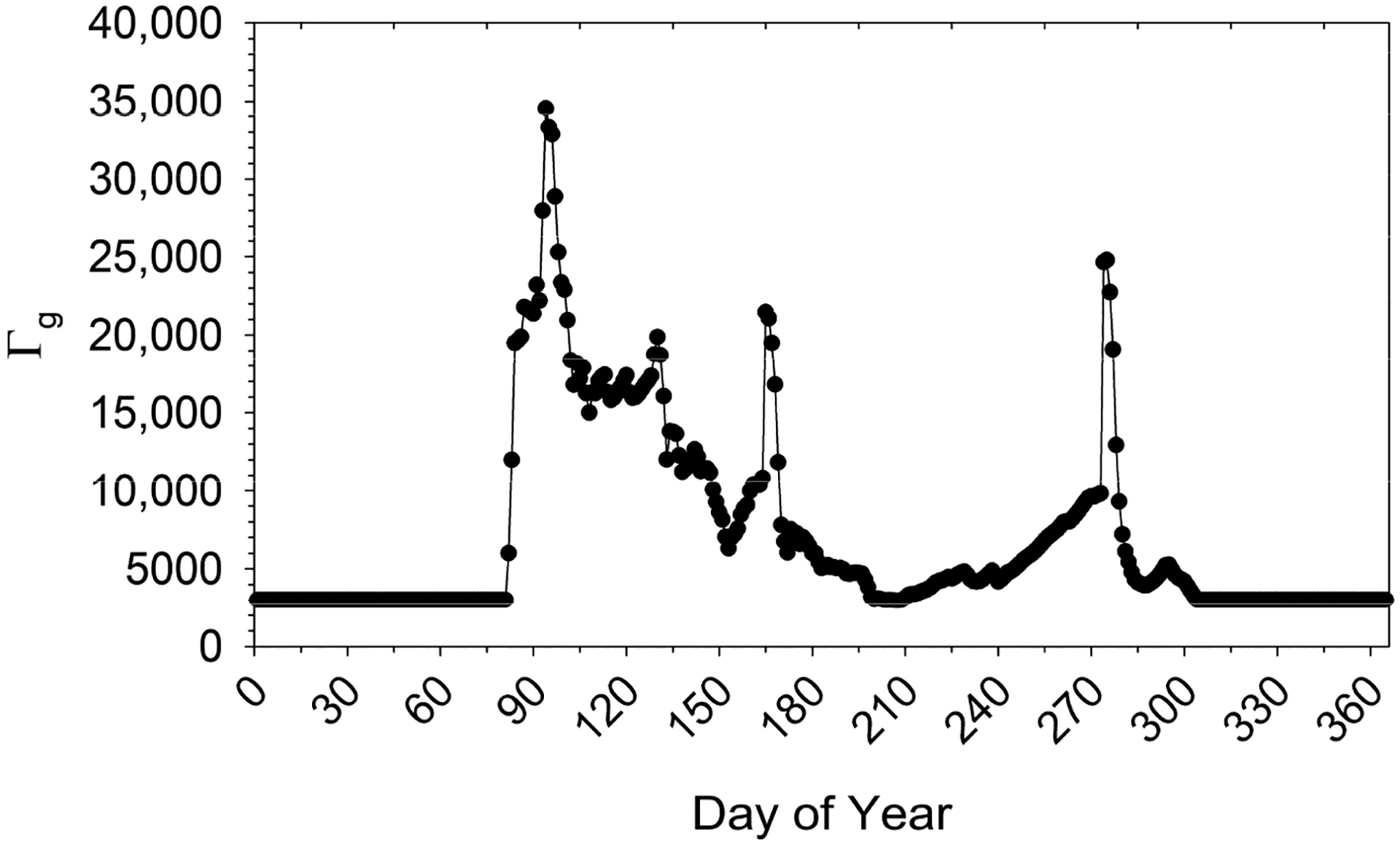
Daily time series of ground NH_3_ emission potential (Γ_g_) for croplands.

To assess the temporal dynamics of Γ_g_ in fertilized soils within the study domain, we examined time series of STAGE-CMAQ estimated Γ_g_ within an area of southern Idaho bounded by 42.0° to 43.0° N and 113.0° to 115.0° W. This produced 125 CMAQ grid cells (12 km) that were then categorized by a pattern of fertilization. The majority of cells indicated three periods of fertilization at approximately DOY 85, 165, and 270, reflecting crop management practices typical of southern Idaho. Cells exhibiting this pattern were grouped, from which a time series of daily median Γ_g_ was determined. Examination of time series for individual CMAQ cells showed similar magnitude for Γ_g_ but pre- and postgrowing season values were substantially larger (~10×) than measurements on fertilized plots described above. STAGE-CMAQ time series generally showed a large increase in Γ_g_ from late fall through winter. Because pH is specified as static, the increase in Γ_g_ indicates accumulation of NH_4_^+^ over time, which could result from an overestimation of mineralization rates or underestimation of nitrification rates. Based on this analysis, the STAGE-CMAQ median daily values were scaled relative to a measured baseline of Γ_g_ = 3000 between the first and last fertilization events with the static baseline value imposed pre- and postgrowing season. The resulting time series of Γ_g_ for croplands used for the in situ STAGE simulations is shown in Figure 3. Variability in Γ_g_ during the growing season (Figure 3) reflects the dynamics of the NH_4_^+^ pool as driven by mineralization, nitrification, NH_3_ loss to the atmosphere, soil moisture, temperature and other factors simulated in STAGE-CMAQ. While the same daily profile of Γ_g_ (Figure 3) is used for all years in the in-situ STAGE simulations presented here, χ_g_ and F_g_ vary hourly, daily, and inter-annually as a function of soil temperature (Equation (9)). This approach incorporates the STAGE-CMAQ model simulated temporal variability of Γ_g_ post-fertilization while anchoring the magnitude to observed baseline soil NH_4_^+^, thereby removing the likely unrealistic STAGE-CMAQ simulated values in fall and winter. As with Γ_s_, Γ_g_ for sites designated as a mix of cropland and grassland (Table 2) was weighted by land use fraction.

## 3. Results and Discussion

### 3.1. Ambient Ammonia Concentrations

**Figure 4.**
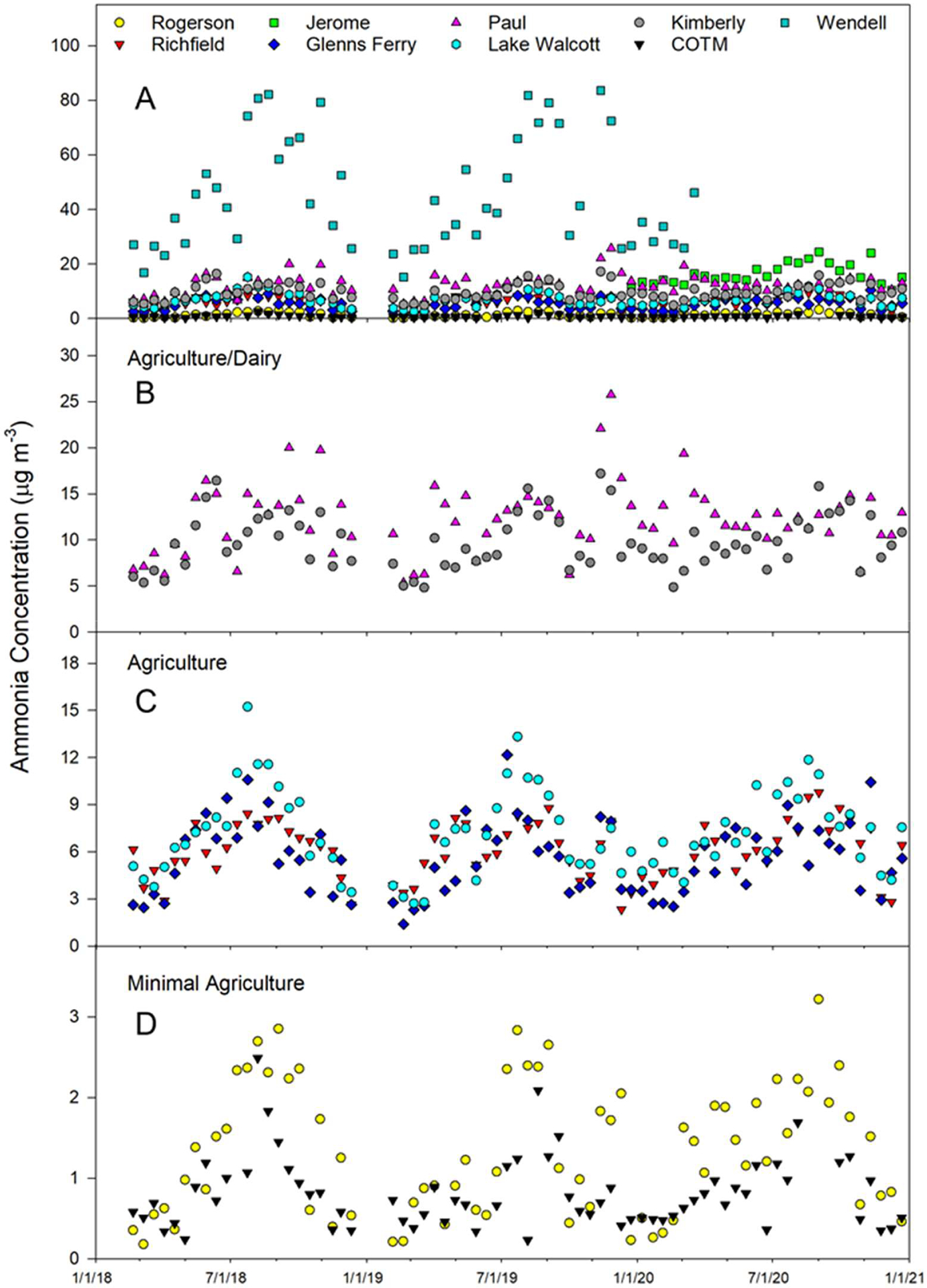
Ambient ammonia concentrations measured over time at each location. Top panel (**A**) includes all data, panel (**B**) consists of sites with both agriculture and dairy present (excluding WE and JE sites, where concentration trends can be viewed in the top panel), panel (**C**) includes sites with influence of agriculture but little dairy influence, and panel (**D**) includes sites with minimal impact due to agriculture and no dairy impact.

The ambient NH_3_ concentrations over the study period by site are shown in Figure 4. All sites followed the general trend of having the lowest concentrations in the winter (December through February) and the greatest concentrations in late summer through fall (July–October), which is consistent with other studies that reported higher NH_3_ concentrations in the hotter months of the year [6,41,42]. The greatest concentrations of NH_3_ were found at the WE site (intensive dairy) and ranged from 15 to 84 μg m^−3^. The JE site (agriculture/dairy) had ambient NH_3_ concentrations that ranged from 10 to 24 μg m^−3^. Sites that were influenced by dairies either in the immediate vicinity or upwind of the location (PA and KI), had NH_3_ concentrations ranging from 5 to 26 μg m^−3^. Sites that were impacted by agriculture but with little to no influence from dairies (GF, RI, and LW) had NH_3_ concentrations ranging from 1.4 to 15 μg m^−3^. The sites located to the far north and south of the Magic Valley, with minimal impact from agriculture and no local dairy influence (RO and COTM), had the lowest concentrations, ranging from 0.2 to 3.2 μg m^−3^. These trends are consistent with other studies that found elevated concentrations of NH_3_ in locations downwind of livestock production facilities [6,43]. Souhar et al. [44] reported ambient NH_3_ concentrations that varied between 2 and 105 μg m^−3^ across a landscape in Bretagne (France) that had a range of agricultural and livestock production intensities. Gradients of NH_3_ concentrations downwind of a swine production facility ranged from 169 μg m^−3^ near the facility edge down to 13.0 μg m^−3^ 698 m downwind due to both dispersion and deposition of NH_3_ to the landscape [45].

To evaluate the influence of climatic variables on ambient NH_3_ concentrations, Spearman correlation analyses were performed with wind speed, air temperature, relative humidity, solar radiation, surface temperature, and soil temperature at 10 cm (Table 4). When all data were combined, solar radiation (r = 0.37), air temperature (r = 0.29), surface temperature (0.29), and soil temperature at 10 cm (r = 0.28) were all positively correlated with NH_3_ concentrations, while relative humidity (r = −0.10) and wind speed (r = −0.60) were negatively correlated. As there was such a large discrepancy in NH_3_ concentrations between sites due to the presence of dairy and agricultural activity, we grouped data into four categories (intensive dairy (WE), agriculture/dairy (KI, PA, JE), agriculture (GF, LW, RI), and minimal agriculture (COTM, RO)) and performed correlations analyses within these groups. At the WE site (intensive dairy), NH_3_ concentration was highly correlated with all meteorological parameters measured with |r| ranging from 0.61 to 0.79, with wind speed and all temperature measurements having the highest correlations. The sites with agriculture/dairy had much weaker correlations |r| = 0.31–0.45 with wind speed having the highest correlation. Both the agriculture and minimal agriculture sites had high correlations with all the temperature measurements, relative humidity, and solar radiation (|r| = 0.59 to 0.72) but a poorer correlation with wind speed (r = −0.20 to −0.38).

Ammonia emissions from dairy cattle housing and manure management have been reported to be significantly positively correlated with wind speed, solar radiation and air temperature and negatively correlated with relative humidity [46–50]. Therefore, daily diurnal variation as well as seasonal variation of NH_3_ emissions from these facilities is common. The WE site, having a large influence of dairy, captured the effects of temperature on NH_3_ emissions from dairy housing and manure storage. Although increasing wind speed enhances NH_3_ emissions from these on farm sources, the dilution effect results in lower NH_3_ concentrations collected at the sampling sites. Sites that were influenced primarily by agriculture had weaker relationships with temperature as NH_3_ emissions from these sources tend to be driven by manure and fertilizer applications [51] which occur in spring when temperatures are cooler while during hotter times of the year, N is utilized by growing crops, therefore reducing available N for loss as NH_3_.

**Figure 5.**
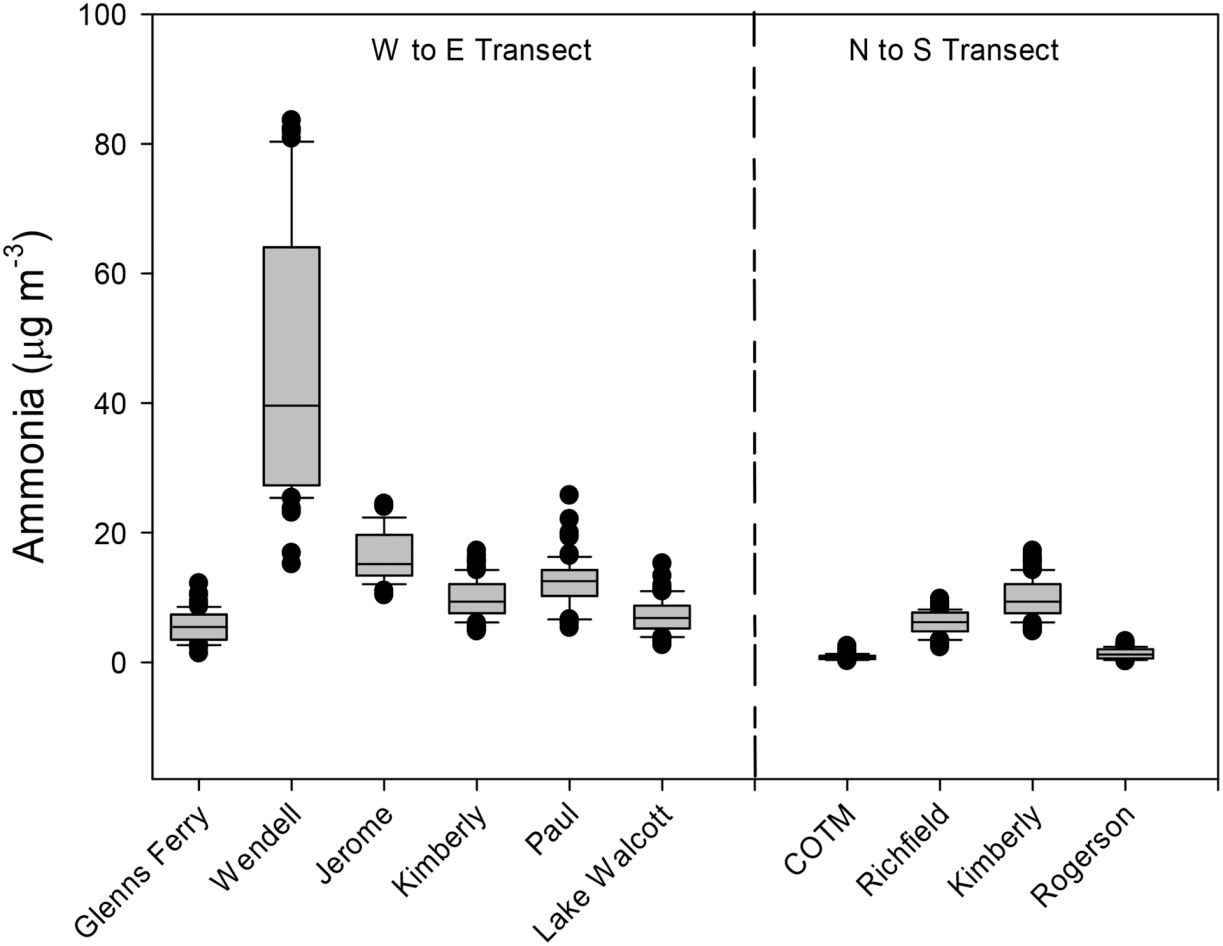
Box plot of ambient ammonia concentration measured over the course of this study across the west-to-east and north-to-south transects. Boxes represent the 25th and 75th percentiles, the line within boxes is the median, the whiskers represent the lower 10% and upper 90% of concentrations, and circles represent outliers.

Average ambient NH_3_ concentrations showed trends across both the west–east and north–south transects (Figure 5). From west to east, average NH_3_ concentrations increased from 5.6 μg m^−3^ at GF to a high of 45.2 μg m^−3^ at WE and then decreased to 7.2 μg m^−3^ at LW, the eastern edge of the Magic Valley. Following a transect from north to south, average NH_3_ concentrations were 0.81 μg m^−3^ at COTM, which should have no impact from agricultural activities, increasing to 9.8 μg m^−3^ at the KI site then decreasing to 1.3 μg m^−3^ in a minimally impacted agricultural area that is dominated by sagebrush steppe (RO).

### 3.2. Ammonia Dry Deposition

**Figure 6.**
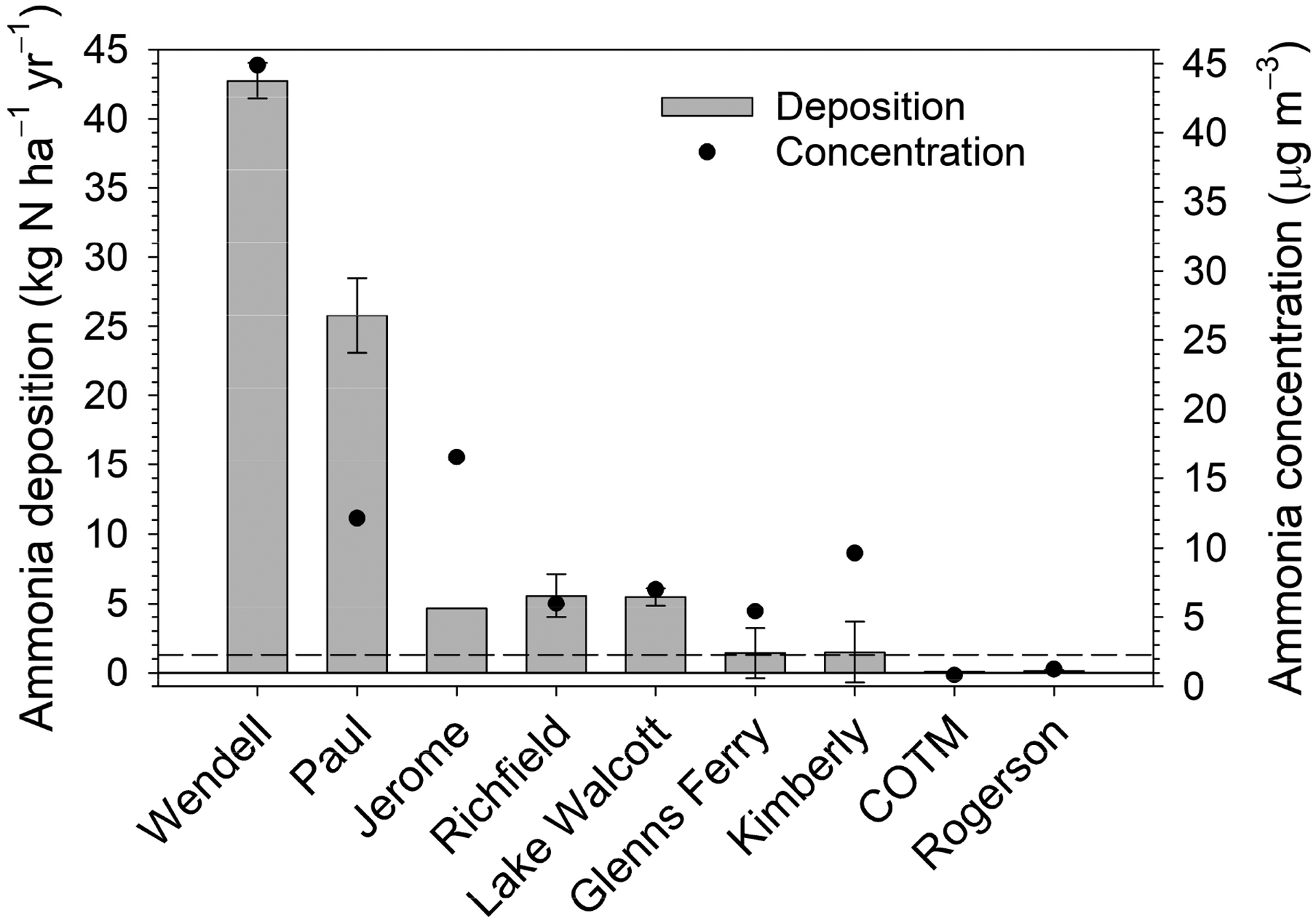
Annual average (2018–2020) ammonia deposition and air concentration across sites. Negative values of deposition indicate net emission. Bars on deposition reflect maximum and minimum values over the three-year period. Dashed line indicates 2018–2020 annual average (1.1 kg N ha^−1^) wet deposition of inorganic nitrogen (NH_4_^+^ + NO_3_^−^) at the COTM NADP/NTN site.

Modeled total annual (2018–2020) average NH_3_ deposition rates and measured concentrations are summarized in Figure 6. Net deposition rates range from 43 kg N ha^−1^ at Wendell to 0.09 kg N ha^−1^ at COTM. There is one National Atmospheric Deposition/National Trends network site within the study region located at COTM, where wet deposition of inorganic N (NH_4_^+^ + NO_3_^−^) averaged 1.1 kg N ha^−1^ over the same three-year period. In general, the modeled dry deposition rate is correlated with ambient air concentration, though relationships between net flux, emission potentials, and LAI impose some variability in this general relationship. For example, Paul has a higher deposition rate than Jerome but a lower average concentration. However, Paul has a much larger peak LAI than Jerome (Figure 2) and thus a much larger surface area for deposition to vegetation.

**Figure 7.**
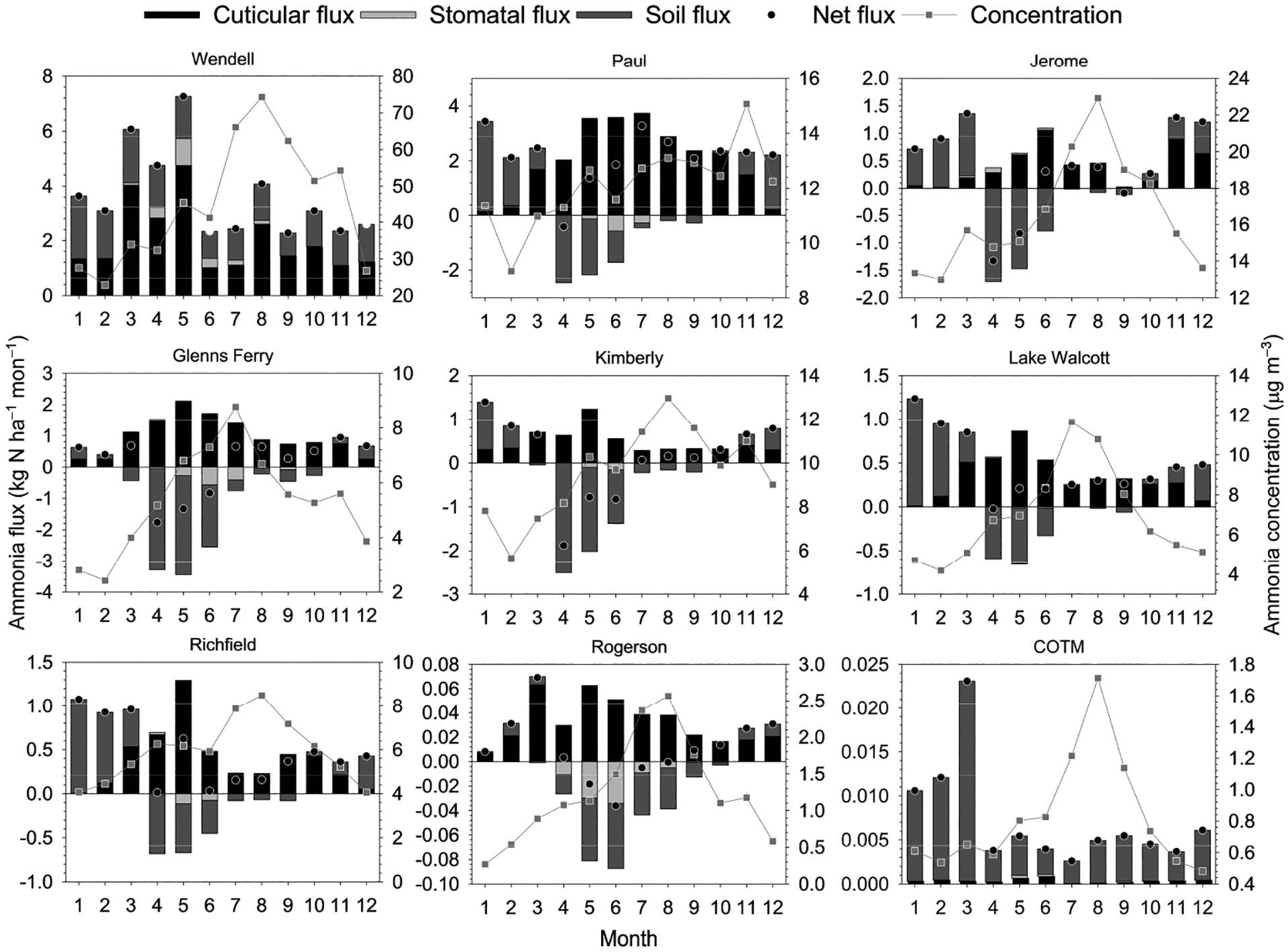
Mean monthly net and component fluxes and air concentrations. Positive values indicate deposition and negative values indicate emission.

Modeled monthly average component and net fluxes are summarized along with measured air concentrations in Figure 7. A general pattern observed across sites is that the peak deposition rate occurs earlier in the year than the peak concentration (summer). During the warmest months, the exponential relationship between temperature and soil/vegetation emission potentials results in larger surface compensation points (Equation (9)) compared to cooler months, causing lower deposition rates or net emission via leaf stomata and the ground (Equations (6) and (8)) and reducing overall net deposition rates. Air concentrations are large enough at WE that net deposition is observed to the cuticle, stomata and ground year-round. A much different pattern is observed at RO, where air concentrations peak at only 2.6 μg NH_3_ m^−3^ in summer. Stomatal and soil compensation points exceed the canopy compensation point and ambient air concentration over much of the spring and summer. Emission from these ecosystem compartments offsets net deposition, resulting in a small annual net deposition flux. Though the air concentration is slightly lower at COTM, deposition is observed year-round via the stomata and soil due to the lower land use weighted emission potential associated with a mostly barren landscape. The importance of LAI, as noted above, is illustrated by the relatively larger cuticular flux at PA (lower air concentration) compared to JE (higher air concentration). Croplands (PA, JE, GF, KI) become a net source of NH_3_ during spring months reflecting increased Γ_g_ and emissions associated with fertilizer application.

### 3.3. Key Model Sensitivities

The relative importance of individual exchange pathways to the total flux, which is calculated here as the sum of the absolute values of the component fluxes, is summarized in Table 5. Note that the modeled total flux determined for this analysis differs from the net flux described in Section 2.4. Overall, exchange via the cuticle surface of the vegetation is the most important pathway, accounting for 61% of the total flux, on average, across sites. This may be expected since the cuticle emission potential (Γ_cut_) is set to 0 in the current configuration of STAGE (i.e., deposition only), whereas bidirectional exchange can occur via the stomata and ground. Exchange with the ground is the next most important pathway, accounting for 35% of the total flux across sites, followed by the stomatal pathway (4%). The exception to this pattern is COTM, which is mostly barren and thus has a very low LAI (Figure 2), resulting in the ground being the primary exchange pathway. Thus, two key model components are the parameterization of the cuticular resistance and the soil emission potential (Γ_g_).

The cuticular resistance for NH_3_ is typically parameterized as a function of LAI, surface wetness, and the amount of NH_3_ dissolved in water residing on the cuticle surface [16] or its pH [52]. The approach used in STAGE is to separate the total cuticular resistance (*R*_*cut*_) into wet periods (*R*_*cut,wet*_) and dry periods (*R*_*cut,wet*_) as:

(16) $$R_{\text{cut}}={\left[LAI\left(\frac{f_{\text{wetleaf}}}{R_{\text{cut},\text{wet}}}+\frac{1-f_{\text{wetleaf}}}{R_{\text{cut},\text{dry}}}\right)\right]}^{-1}$$

where *f*_*wetleaf*_ is the fraction of the canopy that is considered wet. The parameterization used here [27] for *R*_*cut,dry*_ is:

(17) $$R_{\text{cut},\text{dry}}=R_{cut,\text{min}}e^{\alpha_{cut}(100-RH)}$$

As the surfaces are considered dry most of the time, that is, free of macroscale water layers associated with liquid precipitation, dew, and guttation, here we explore the sensitivity of the cuticular flux (*F*_*cut*_) to *R*_*cut,dry*_. The cuticular resistance cannot be measured directly; rather, it is typically inferred from nighttime canopy-scale NH_3_ flux measurements under the assumption that the stomatal flux pathway is closed and the ground flux is negligible [27]. Such datasets show a clear, generally non-linear, relationship with RH, indicating a reduction in *R*_*cut,dry*_ as microscale water layers form on the cuticle surface [27] at high RH. The minimum cuticular resistance (*R*_*cut,min*_) demonstrates a relationship with pH of the cuticle surface water, parameterized as the ratio of total acid to NH_3_ in the atmosphere (i.e., acid ratio, [14]). Here, we assume an acid ratio of 0.5, yielding *R*_*cut,min*_ = 63 [27]. An empirical factor (*α*_*cut*_) defines the form of the exponential relationship between *R*_*cut,dry*_ and RH, thus exerting important control on the dynamics of *F*_*cut*_.

Based on a metanalysis of existing datasets, Massad et al. [27] separate *α*_*cut*_ by ecosystem type based on the expectation that the factors controlling the relationship between RH and formation of microscale water layers on the cuticle, such as hygroscopicity and aerosol uptake, will differ by plant species [27]. Values used in our analysis include grassland and arable land use types, for which the mean and standard deviation of *α*_*cut*_ reported by Massad et al. [27] are 0.176 ± 0.126 and 0.148 ± 0.113, respectively. Using the reported standard deviations to guide a simple sensitivity test, decreasing *α*_*cut*_ by 0.1, thereby decreasing *R*_*cut,dry*_, increases annual deposition by ~30% across sites. Our results reinforce findings from other work that additional process-level flux measurements are needed to better constrain this parameter and the cuticular resistance in general [5,27].

As described above, the sorption characteristics of the soil determine the fraction of soil total NH_4_^+^ that resides in solution and is therefore available for exchange with the atmosphere as NH_3_. Sorption characteristics have been shown to vary widely by soil type, but studies generally point to the important roles of cation exchange capacity (CEC) and clay content [33]. The sorption characteristics of the soil sampled in our study predict that the vast majority of NH_4_^+^ is adsorbed to the soil matrix and that only ~1% of total soil NH_4_^+^ will be in solution. Taking Glenns Ferry as an example of a “cropland” site, the cumulative emission of NH_3_ from the soil during the period in which Γ_g_ is elevated above baseline due to fertilization (day of year (DOY) 80 to 300 in Figure 3) is equivalent to ~10.9 kg N ha^−1^. This emission rate corresponds to 7.6% of a typical N application rate of 144 kg N ha^−1^ y^−1^ for croplands in our study area [11]. The fraction of NH_3_ typically emitted from fertilizer applications ranges from 3% to >65% ([11] and references therein) depending on soil and fertilizer type, application method, meteorology, and soil conditions at the time of application. Within the region, fertilizer (mainly urea) is typically incorporated into the soil following application and then irrigated within a short period of time, further incorporating the fertilizer and reducing NH_3_ emissions, and therefore we would expect losses to be at the lower end of the range. Adjusting the sorption parameters to increase the fraction of NH_4_^+^ in solution by a factor of 10 results in an equivalent cumulative soil emission of 128 kg N ha^−1^, or 89% of the typical N application rate. In a recent analysis, Pleim et al. [8] parameterized the available NH_4_^+^ fraction in the CMAQv5.3 M3Dry deposition option using a function of CEC and noted that the resulting large fluxes for high pH agricultural soils warrant further study. Pleim et al. [8] also noted that studies in which sorption characteristics are determined from models applied to equilibrium experiments stress the dependence on specific soil conditions and difficulty in generalizing the results. The modeled fluxes presented here are clearly sensitive to the parameterization of the soil sorption characteristics and the assumptions underlying the adsorption equilibrium concept. While the sensitivity analysis here focuses on sorption characteristics, it is acknowledged that resistances along the ground exchange pathway (i.e., *R*_*inc*_, *R*_*bg*_, and *R*_*soil*_) remain poorly understood for some conditions, and thereby contribute additional uncertainty to the ground and net canopy-scale fluxes.

### 3.4. Comparison of In-Situ and CMAQ Air Concentrations and Deposition

The U.S. EPA Office of Atmospheric Protection generates annual total deposition (TDep) maps for a variety of chemical species using a measurement-model fusion (MMF) technique [53]. TDep MMF combines measurements and spatial interpolation of wet deposition from the National Atmospheric Deposition Program (NADP)/National Trends Network, air concentration measurements from the Clean Air Status and Trends Network (CASTNET), and output from CMAQv5.3.2 [17], which incorporates the STAGE model to simulate bidirectional exchange of NH_3_. For measured species, observations of air concentration are used to bias correct the CMAQ deposition output. For unmeasured species, both air concentrations and dry deposition are simulated using CMAQ [17]. For TDep, CMAQ simulations are output at a grid size of 12 km × 12 km and re-gridded to 4 km by 4 km to match the spatially interpolated wet deposition. TDep maps and gridded data are provided to the public via the NADP (https://nadp.slh.wisc.edu/committees/tdep/, Accessed on 15 December 2023).

**Figure 8.**
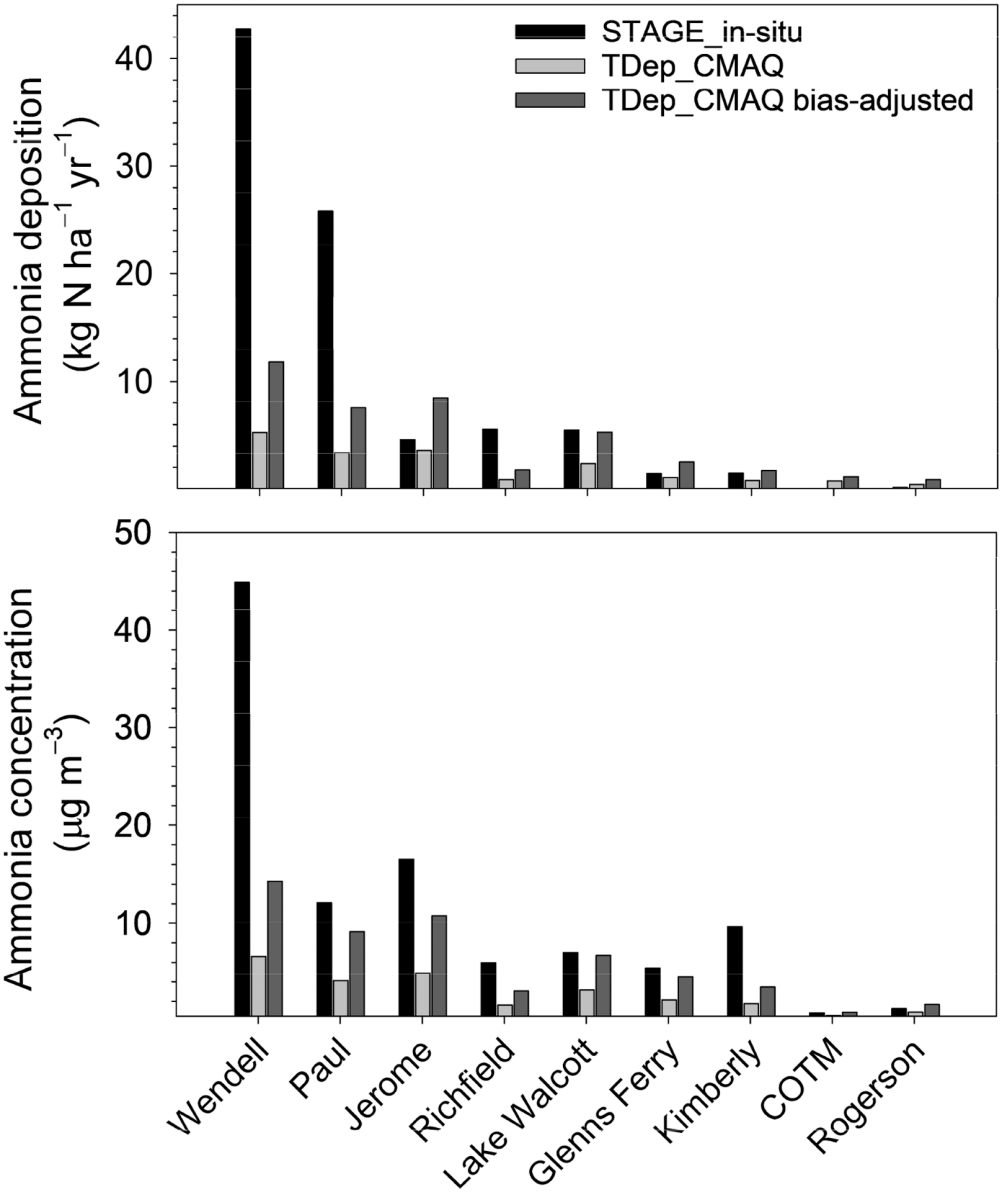
Mean annual STAGE ammonia deposition estimates (**top**) and in situ measured air concentrations (**bottom**) versus TDep deposition with concentrations from CMAQ (TDep_CMAQ) or with concentrations bias-adjusted using AMoN measurements (TDep_CMAQ bias-adjusted).

The current TDep MMF methodology does not bias-correct NH_3_ concentrations or, therefore, dry deposition. However, estimates of deposition derived from air concentrations bias corrected using NH_3_ observations from the NADP AMoN have recently been generated, as for other species following Schwede and Lear [53], in an effort to extend the current methodology. In Schwede and Lear [53], the bias correction is species-dependent and is related to the radius around a monitoring location within which air concentrations are spatially correlated beyond a standard threshold. This “maximum radius of influence” is defined here as 1000 km for NH_3_, which will be refined through ongoing, more detailed analysis of the spatial correlation between NH_3_ measurements at the continental scale and better understanding of the relationship between NH_3_ air concentration and bidirectional exchange. Differences in deposition estimates between the in situ and CMAQ models could therefore reflect the net result of differences in air concentrations, parameterization of the air–surface exchange process (i.e., surface emission potentials), meteorology that affects surface characteristics and atmospheric resistances, and land use characteristics within the 1 km radius of the site considered in the in-situ modeling approach versus the 12 km CMAQ grid. Ammonia fluxes and concentrations from the in-situ measurement and the STAGE inferential modeling approach presented above and TDep_CMAQ estimates with and without bias correction are presented in Figure 8. Note that the CMAQ bias correction procedure only incorporates sites from the AMoN network.

At all sites other than COTM and RO, in situ STAGE deposition estimates (black bars in Figure 8) are larger than TDep_CMAQ estimates (light gray), particularly at sites where high deposition rates occur. At Wendell, in situ estimated deposition is ~8× larger than estimated by TDep_CMAQ. The difference in deposition rates corresponds to differences in air concentration, shown in the lower panel of Figure 8. The large differences in air concentration are likely related to an underestimate of agricultural NH_3_ emissions in CMAQ but may also relate to model treatment of boundary layer dynamics, transport, and differences in the values used for NH_3_ emission potentials that contribute to bidirectional exchange. Bias adjustment using AMoN observations (dark gray bars in Figure 8) increases the TDep_CMAQ-modeled air concentration and deposition rates, bringing both into better agreement with in situ deposition estimates and observed concentrations and further illustrating the general relationship between deposition rate and air concentration.

TDep_CMAQ deposition estimates (default and bias-adjusted) exceed in-situ model estimates at sites with the lowest concentrations (COTM, RO). However, in situ model estimates of dry deposition remain larger than the bias adjusted TDep_CMAQ deposition estimates at some of the sites with higher concentrations (WE, PA, RI, LW). The difference between the in-situ and bias-adjusted TDep_CMAQ modeled deposition estimates over a gradient in concentration (high vs. low) may partly reflect the role of enhanced specificity in model inputs, especially the emission potentials, in the in-situ model. Taking COTM and RO as examples, TDep uses the CMAQ default Γ_g_ of 20 [8] (for unfertilized soils, while the in-situ model uses a Γ_g_ of 900 based on measurements (see Section 2.9). The in-situ Γ_s_ for natural vegetation (685) is also larger than specified in CMAQv5.3 (~250). The higher emission potentials for the in-situ model implies a higher compensation point, resulting in a smaller downward concentration gradient from the atmosphere to the surface (COTM) and lower threshold for stomatal and soil emissions (RO, Figure 7). This pattern is consistent with the lower in-situ net deposition estimates at these low concentration sites compared to the TDep_CMAQ estimates. While differences in meteorology or other land use characteristics between TDep_CMAQ and in-situ modeled deposition estimates were not specifically assessed, implementing or bias-adjusting measured air concentrations and use of site-specific emission potentials are influential among the model estimates.

## 4. Conclusions

The influence of dairy production on NH_3_ concentrations across the valley can clearly be seen, with the highest concentrations in areas with high dairy density. Peak NH_3_ concentrations were 4–5 times greater at the intensive dairy site compared to mixed agriculture/dairy or agricultural sites, and 26 times greater than minimal agricultural sites. Temperature was one of the larger drivers of emissions and therefore seasonal trends were prominent at all sites.

Elevated NH_3_ concentrations downwind of dairies result in high rates of NH_3_ dry deposition to the landscape. Dry deposition rates estimated using an in-situ bidirectional exchange model predict net downward annual fluxes spanning three orders of magnitude across the range of observed air concentrations. Our results indicate that annual dry deposition rates in areas of intensive dairy production can approach 45 kg N ha^−1^ y^−1^, compared to < 1 kg N ha^−1^ y^−1^ in natural landscapes absent of dairy production and other agriculture. Among the sites studied, croplands are net sources of NH_3_ to the atmosphere after fertilization, particularly during the spring, but take up NH_3_ from the atmosphere during other periods.

Comparison of dry deposition fluxes modeled using measured NH_3_ concentrations to estimates derived from regional chemical transport modeling highlights the utility of spatially dense monitoring of NH_3_ in agricultural areas. Expansion of NADP/AMoN in agricultural areas is needed to better characterize spatial and temporal variability of NH_3_ for more extensive evaluation of gridded chemical transport models [54,55], to support assessments of deposition and air quality and to inform sub-grid model processes. Sensitivity testing of the field-scale (i.e., in-situ) bidirectional modeling framework reveals the need for better understanding of the factors controlling the soil emission potential in environments with high soil pH. Measurements of air-surface exchange fluxes coupled with temporally resolved measurements of soil chemistry are needed to more rigorously test the sorption modeling framework for predicting soil NH_3_ compensation points. Until now, treatment of soil flux processes in NH_3_ exchange modeling of natural landscapes has received less attention than parameterization of vegetation exchange pathways. A direction of research toward better understanding soil processes is needed to improve understanding of the importance of NH_3_ dry deposition to arid and sparsely vegetated natural ecosystems across the western U.S.

Our results suggest that the natural sagebrush steppe landscapes interspersed within and surrounding agricultural areas in the Magic Valley receive NH_3_ dry deposition rates within and above the range of critical loads (3–8.4 kg N ha^−1^ y^−1^) reported by Pardo et al. [12] for North American deserts. Consequences can include changes in plant community structure, including increases in biomass of invasive grass species [56], and increased soil water use [57]. Increases in wildfire size and frequency in arid and semiarid ecosystems of the western U.S. have been associated with expansion of non-native grasses such as cheatgrass (*Bromus tectorum*) [58], now common to sagebrush ecosystems in southern Idaho. Such changes in fire patterns favor further expansion of invasive grasses and associated loss of native plants less tolerant of shorter fire intervals. While our results inform the importance of NH_3_ dry deposition relative to current critical loads, additional work is needed to improve understanding of the role of N deposition in the health of sagebrush ecosystems, taking into account interactions between disturbance, N inputs, and changing climate.

## Figures and Tables

**Table 1.** Study site ID and name, location, elevation, site type, and site description.

ID	Site Name	Latitude	Longitude	Elevation	Deployment Dates	Site Type	Site Description
		Decimal Degrees		m			
COTM	COTM	43.44784	−113.55174	1798	2/5/2018–12/22/2020	Minimal agriculture	Lava fields, sagebrush steppe
GF	Glenns Ferry	42.94426	−115.33367	772	2/5/2018–12/22/2020	Agriculture	Irrigated agriculture, sagebrush steppe
JE	Jerome	42.72248	−114.51163	1159	12/23/2019–12/22/2020	Agriculture/dairy	Moderate density dairy, urban
KI	Kimberly	42.55297	−114.35493	1185	2/5/2018–12/22/2020	Agriculture/dairy	Irrigated agriculture, few dairies
LW	Lake Walcott	42.67184	−113.49472	1266	2/5/2018–12/22/2020	Agriculture	Sagebrush steppe, some irrigated agriculture
PA	Paul	42.59545	−113.87393	1281	2/5/2018–12/22/2020	Agriculture/dairy	Irrigated agriculture, some dairy
RI	Richfield	43.05461	−114.16023	1317	2/5/2018–12/22/2020	Agriculture	Irrigated agriculture, some dairy
RO	Rogerson	42.15674	−114.69247	1607	2/5/2018–12/22/2020	Minimal agriculture	Sagebrush steppe
WE	Wendell	42.72	−114.69366	1034	2/5/2018–3/17/2020	Intensive dairy	High-density dairy, irrigated agriculture

**Table 2.** Land use types within a 1 km radius of each site, vegetation emission potential (Γ_s_) and ground emission potential (Γ_g_).

Site	Land Use Type (Fraction)	Γ_s_	Γ_g_
Glenns Ferry	Croplands	4750	Variable
Kimberly	Croplands	4750	Variable
COTM	Barren or sparsely vegetated (0.87), grasslands (0.13)	149	117
Rogerson	Grasslands	1145	900
Richfield	Grasslands (0.5), croplands (0.5)	2947	Variable
Wendell	Grasslands (0.8), croplands (0.2)	1866	Variable
Paul	Croplands	4750	Variable
Lake Walcott	Grasslands (0.58), croplands (0.42)	2659	Variable
Jerome	Croplands	4750	Variable

**Table 3.** Sources of meteorological data for each site. Variables include precipitation rate (*P*_*recip*_), relative humidity (*RH*), air temperature (*T*_*a*_), surface wetness (*SW*), atmospheric pressure (*P*_*a*_), soil moisture (*SM*), soil temperature (*T*_*soil*_), wind speed (*WS*), friction velocity (u*), and downward shortwave radiation (*R*_*g_in*_). O—observation from meteorological station; M1—RTMA (2.5 × 2.5 km); M2—NLDAS (12 × 12 km); M3—Noah LSM. Within a distance of 20 km, measurements from a meteorological station are not available for COTM and Rogerson.

Site	Meteo. Station (Distance in km)	*T*_*a*_	*RH*	*WS*	*P*_*a*_	*R*_*g_in*_	*P*_*recip*_	u*	*SW*	*T*_*soil*_	*SM*
Glenns Ferry	GFRI (8.8)	O	O	O	M1	O	O	M3	M3	M3	M3
Kimberly	TWFI (1.1)	O	O	O	M1	O	O	M3	M3	M3	M3
COTM	N.A.	M1	M1	M1	M1	M2	M2	M3	M3	M3	M3
Rogerson	N.A.	M1	M1	M1	M1	M2	M2	M3	M3	M3	M3
Richfield	ICHI (2.2)	O	O	O	M1	O	M2	M3	M3	M3	M3
Wendell	TFGI (18.4)	O	O	O	M1	O	O	M3	M3	M3	M3
Paul	RPTI (0.1)	O	O	O	M1	O	O	M3	M3	M3	M3
Lake Walcott	MDKI (17.3)	O	O	O	O	O	M2	M3	M3	M3	M3
Jerome	TFGI (18.6)	O	O	O	M1	O	O	M3	M3	M3	M3

**Table 4.** Spearman correlation of ambient ammonia concentration with climatic variables for all sites combined and for sites delineated by the extent of agriculture and dairy influence. (Data from 20 February 2018 through 17 March 2020, correlation coefficients with their respective *p* value immediately below are presented). Site types/groups are defined in Table 1.

Climatic Variables						
	Wind Speed	Air Temperature	Relative Humidity	Soil Temperature 10 cm	Surface Temperature	Solar Radiation
	m/s	K	%	K	K	W/m^2^
All data	−0.60 <0.0001	0.29 <0.0001	−0.10 0.014	0.28 <0.0001	0.29 <0.0001	0.37 <0.0001
WE (intensive dairy)	−0.79 <0.0001	0.71 <0.0001	−0.63 <0.0001	0.70 <0.0001	0.69 <0.0001	0.61 <0.0001
Agriculture/dairy	−0.45 <0.0001	0.35 <0.0001	−0.31 <0.0001	0.34 <0.0001	0.33 <0.0001	0.32 <0.0001
Agriculture	−0.20 0.003	0.70 <0.0001	−0.66 <0.0001	0.71 <0.0001	0.71 <0.0001	0.62 <0.0001
Minimal agriculture	−0.38 <0.0001	0.72 <0.0001	−0.70 <0.0001	0.68 <0.0001	0.67 <0.0001	0.59 <0.0001

**Table 5.** Percent contribution of component pathways to total annual flux calculated as the sum of the absolute values of the component fluxes.

	% Stomatal	% Cuticular	% Ground	Land Use
Glenns Ferry	6.9	52.0	41.1	Croplands
Jerome	3.5	89.5	7.0	Croplands
Kimberly	2.7	55.2	42.1	Croplands
Paul	3.4	83.6	13.0	Croplands
Lake Walcott	0.3	76.3	23.4	Croplands/grasslands
Richfield	2.2	71.9	25.9	Croplands/grasslands
Wendell	5.4	53.8	40.8	Croplands/grasslands
COTM	1.3	5.2	93.5	Grassland/barren
Rogerson	14.1	60.6	25.3	Grasslands

## Data Availability

The data presented in this study are available on request from the corresponding author. The data are not publicly available due to restrictions.

## References

[1] USEPA. 2020 NEI Supporting Data and Summaries; United States Environmental Protection Agency: Washington, DC, USA, 2020.

[2] Theobald MR; Bealey WJ; Tang YS; Vallejo A; Sutton MA A simple model for screening the local impacts of atmospheric ammonia. Sci. Total Environ 2009, 407, 6024–6033. 19765803 10.1016/j.scitotenv.2009.08.025

[3] Kelleghan DB; Hayes ET; Everard M; Curran TP Predicting atmospheric ammonia dispersion and potential ecological effects using monitored emission rates from an intensive laying hen facility in Ireland. Atmos. Environ 2021, 247, 118214.

[4] Stevens CJ; Tilman D Point source ammonia emissions are having a detrimental impact on prairie vegetation. Water Air Soil Pollut. 2010, 211, 435–441.

[5] Walker JT; Beachley G; Amos HM; Baron JS; Bash J; Baumgardner R; Bell MD; Benedict KB; Chen X; Clow DW; Toward the improvement of total nitrogen deposition budgets in the United States. Sci. Total. Environ 2019, 691, 1328–1352. 31466212 10.1016/j.scitotenv.2019.07.058 PMC7724633

[6] Allen AG; Harrison RM; Wake MT A meso-scale study of the behaviour of atmospheric ammonia and ammonium. Atmos. Environ. 1967 1988, 22, 1347–1353.

[7] Baker J; Battye WH; Robarge W; Pal Arya S; Aneja VP Modeling and measurements of ammonia from poultry operations: Their emissions, transport, and deposition in the Chesapeake Bay. Sci. Total Environ 2020, 706, 135290. 31838459 10.1016/j.scitotenv.2019.135290

[8] Pleim JE; Ran L; Appel W; Shephard MW; Cady-Pereira K New bidirectional ammonia flux model in an air quality model coupled with an agricultural model. J. Adv. Model. Earth Syst 2019, 11, 2934–2957. 33747353 10.1029/2019MS001728 PMC7970535

[9] USDA-ERS. Dairy Production Background; USDA: Washington, DC, USA, 2022.

[10] USDA-NASS. Idaho Statistics; USDA: Washington, DC, USA, 2023.

[11] Leytem AB; Williams P; Zuidema S; Martinez A; Chong YL; Vincent A; Vincent A; Cronan D; Kliskey A; Wulfhorst JD; Cycling phosphorus and nitrogen through cropping systems in an intensive dairy production region. Agronomy 2021, 11, 1005.

[12] Pardo LH; Fenn ME; Goodale CL; Geiser LH; Driscoll CT; Allen EB; Baron JS; Bobbink R; Bowman WD; Clark CM; Effects of nitrogen deposition and empirical nitrogen critical loads for ecoregions of the United States. Ecol. Appl 2011, 21, 3049–3082.

[13] Walker JT; Beachley G; Zhang L; Benedict KB; Sive BC; Schwede DB A review of measurements of air-surface exchange of reactive nitrogen in natural ecosystems across North America. Sci. Total Environ 2020, 698, 133975. 31499348 10.1016/j.scitotenv.2019.133975 PMC7032654

[14] Nemitz E; Milford C; Sutton M A two-layer canopy compensation point model for describing bi-directional biosphere-atmosphere exchange of ammonia. Q. J. R. Meteorol. Soc 2001, 127, 815–833.

[15] Zhang L; Wright LP; Asman WAH Bi-directional air-surface exchange of atmospheric ammonia: A review of measurements and a development of a big-leaf model for applications in regional-scale air-quality models. J. Geophys. Res. Atmos 2010, 115, D20.

[16] Pleim JE; Bash JO; Walker JT; Cooter EJ Development and evaluation of an ammonia bidirectional flux parameterization for air quality models. J. Geophys. Res. Atmos 2013, 118, 3794–3806.

[17] Appel KW; Bash JO; Fahey KM; Foley KM; Gilliam RC; Hogrefe C; Hutzell WT; Kang D; Mathur R; Murphy BN; The Community Multiscale Air Quality (CMAQ) model versions 5.3 and 5.3.1: System updates and evaluation. Geosci. Model Dev 2021, 14, 2867–2897. 34676058 10.5194/gmd-14-2867-2021 PMC8525427

[18] Lee HM; Paulot F; Henze DK; Travis K; Jacob DJ; Pardo LH; Schichtel BA Sources of nitrogen deposition in Federal Class I areas in the US. Atmos. Chem. Phys 2016, 16, 525–540.

[19] Clark CM; Phelan J; Doraiswamy P; Buckley J; Cajka JC; Dennis RL; Lynch J; Nolte CG; Spero TL Atmospheric deposition and exceedances of critical loads from 1800−2025 for the conterminous United States. Ecol. Appl 2018, 28, 978–1002. 29714821 10.1002/eap.1703 PMC8637495

[20] Benish SE; Bash JO; Foley KM; Appel KW; Hogrefe C; Gilliam R; Pouliot G Long-term regional trends of nitrogen and sulfur deposition in the United States from 2002 to 2017. Atmos. Chem. Phys 2022, 22, 12749–12767. 10.5194/acp-22-12749-2022 PMC11864274 40012769

[21] Makar PA; Akingunola A; Aherne J; Cole AS; Aklilu YA; Zhang J; Wong I; Hayden K; Li SM; Kirk J; Estimates of exceedances of critical loads for acidifying deposition in Alberta and Saskatchewan. Atmos. Chem. Phys 2018, 18, 9897–9927.

[22] Zhang L; Vet R; O’Brien J; Mihele C; Liang Z; Wiebe A Dry deposition of individual nitrogen species at eight Canadian rural sites. J. Geophys. Res 2009, 114, D02301.

[23] Flechard CR; Nemitz E; Smith RI; Fowler D; Vermeulen AT; Bleeker A; Erisman JW; Simpson D; Zhang L; Tang YS; Dry deposition of reactive nitrogen to European ecosystems: A comparison of inferential models across the NitroEurope network. Atmos. Chem. Phys 2011, 11, 2703–2728.

[24] Sigma-Aldrich. Radiello Manual, Sigma-Aldrich: St. Louis, MO, USA.

[25] Clifton OE; Schwede D; Hogrefe C; Bash JO; Bland S; Cheung P; Coyle M; Emberson L; Flemming J; Fredj E; A single-point modeling approach for the intercomparison and evaluation of ozone dry deposition across chemical transport models (Activity 2 of AQMEII4). Atmos. Chem. Phys 2023, 23, 9911–9961. 37990693 10.5194/acp-23-9911-2023 PMC10659075

[26] Walker JT; Chen X; Wu Z; Schwede D; Daly R; Djurkovic A; Oishi AC; Edgerton E; Bash J; Knoepp J; Atmospheric deposition of reactive nitrogen to a deciduous forest in the southern Appalachian Mountains. Biogeosciences 2023, 20, 971–995. 39434786 10.5194/bg-20-971-2023 PMC11492993

[27] Massad RS; Nemitz E; Sutton MA Review and parameterisation of bi-directional ammonia exchange between vegetation and the atmosphere. Atmos. Chem. Phys 2010, 10, 10359–10386.

[28] Søren H; Jan Kofod S Apoplastic pH and Ammonium Concentration in Leaves of Brassica napus L. Plant Physiol. 1995, 109, 1453–1460. 12228682 10.1104/pp.109.4.1453 PMC157681

[29] Foley KM; Pouliot GA; Eyth A; Aldridge MF; Allen C; Appel KW; Bash JO; Beardsley M; Beidler J; Choi D; 2002–2017 anthropogenic emissions data for air quality modeling over the United States. Data Brief 2023, 47, 109022. 36942100 10.1016/j.dib.2023.109022 PMC10023994

[30] Chen F; Dudhia J Coupling an advanced land surface-hydrology model with the Penn State-NCAR MM5 modeling system. part I, model implementation and sensitivity. Mon. Weather. Rev 2001, 129, 569–585.

[31] Chen F; Janjic Z; Mitchell K Impact of atmospheric surface layer parameterization in the new land-surfaces cheme of the NCEP mesoscale Eta numerical model. Bound. Layer Meteorol 1997, 185, 391–421.

[32] Walker J; Daly R; Djurkovic A; Barnes M; Baumgardner R; Macy T; Puchalski M; Isil S; Mishoe K; Stewart M; AMoN Site Characterization Study for NH3 Bidirectional Flux Modeling: Phase I Field Measurements; U.S. Environmental Protection Agency: Washington, DC, USA, 2023.

[33] Vogeler I; Cichota R; Snow VO; Dutton T; Daly B Pedotransfer functions for estimating ammonium adsorption in soils. Soil Sci. Soc. Am. J 2011, 75, 324–331.

[34] Venterea RT; Clough TJ; Coulter JA; Breuillin-Sessoms F; Wang P; Sadowsky MJ Ammonium sorption and ammonia inhibition of nitrite-oxidizing bacteria explain contrasting soil N_2_O production. Sci. Rep 2015, 5, 12153. 26179972 10.1038/srep12153 PMC4503984

[35] Hinz C Description of sorption data with isotherm equations. Geoderma 2001, 99, 225–243.

[36] Sieczka A; Koda E Kinetic and equilibrium studies of sorption of ammonium in the soil-water environment in agricultural areas of central poland. Appl. Sci 2016, 6, 296.

[37] Liu Y; Shen L From langmuir kinetics to first- and second-order rate equations for adsorption. Langmuir 2008, 24, 11625–11630. 18788769 10.1021/la801839b

[38] Cooter EJ; Bash JO; Benson V; Ran L Linking agricultural crop management and air quality models for regional to national-scale nitrogen assessments. Biogeosciences 2012, 9, 4023–4035.

[39] Williams JR The EPIC model. In Computer Models of Watershed Hydrology; Singh VP, Ed.; Water Resources Publ.: Highlands Ranch, CO, USA, 1995; pp. 909–1000.

[40] Bash JO; Cooter EJ; Dennis RL; Walker JT; Pleim JE Evaluation of a regional air-quality model with bidirectional NH_3_ exchange coupled to an agroecosystem model. Biogeosciences 2013, 10, 1635–1645.

[41] Liu P; Ding J; Ji Y; Xu H; Liu S; Xiao B; Jin H; Zhong X; Guo Z; Wang H; Satellite support to estimate livestock ammonia emissions: A Case Study in Hebei, China. Atmosphere 2022, 13, 1552.

[42] Saha CK; Ammon C; Berg W; Fiedler M; Loebsin C; Sanftleben P; Brunsch R; Amon T Seasonal and diel variations of ammonia and methane emissions from a naturally ventilated dairy building and the associated factors influencing emissions. Sci. Total Environ 2014, 468–469, 53–62. 10.1016/j.scitotenv.2013.08.015 24012895

[43] Mielcarek-Bocheńska P; Rzeźnik W Ammonia emission from livestock production in Poland and its regional diversity, in the years 2005–2017. Arch. Environ. Prot 2019, 45, 114–121.

[44] Souhar O; Fauvel Y; Flechard C Measuring and modeling atmospheric ammonia from agricultural sources at a landscape scale. Environ. Eng. Sci 2022, 39, 673–684.

[45] Walker J; Spence P; Kimbrough S; Robarge W Inferential model estimates of ammonia dry deposition in the vicinity of a swine production facility. Atmos. Environ 2008, 42, 3407–3418.

[46] Cassel T; Ashbaugh L; Flocchini R Ammonia flux from open-lot dairies: Development of measurement methodology and emission factors. J. Air Waste Manag. Assoc 2005, 55, 816–825. 16022419 10.1080/10473289.2005.10464659

[47] Grant RH; Boehm MT; Heber AJ Ammonia emissions from anaerobic waste lagoons at pork production operations: Influence of climate. Agric. For. Meteorol 2016, 228–229, 73–84.

[48] Guo H; Hao H; Zhang Q; Wang J; Liu J Components and dispersion characteristics of organic and inorganic odorous gases in a large-scale dairy farm. J. Air Waste Manag. Assoc 2019, 69, 717–725. 30576261 10.1080/10962247.2018.1562389

[49] Lovanh N; Quintanar A; Rysz M; Loughrin J; Mahmood R Effect of Heat Fluxes on Ammonia Emission from Swine Waste Lagoon Based on Neural Network Analyses. J. Environ. Sci. Technol 2014, 7, 16–29.

[50] Wu C; Yang F; Brancher M; Liu J; Qu C; Piringer M; Schauberger G Determination of ammonia and hydrogen sulfide emissions from a commercial dairy farm with an exercise yard and the health-related impact for residents. Environ. Sci. Pollut. Res 2020, 27, 37684–37698. 10.1007/s11356-020-09858-y PMC7496066 32608005

[51] Hojito M; Hayashi K; Matsuura S net Ammonia exchange on grasslands in an intensive dairying region in central Japan. Soil Sci. Plant Nutr 2010, 56, 503–511.

[52] Van Hove LWA; Adema EH; Vredenberg WJ; Pieters GA A study of the adsorption of NH_3_ and SO_2_ on leaf surfaces. Atmos. Environ. 1967 1989, 23, 1479–1486.

[53] Schwede DB; Lear GG A novel hybrid approach for estimating total deposition in the United States. Atmos. Environ 2014, 92, 207–220.

[54] Zhu S; Wu K; Nizkorodov SA; Dabdub D Modeling reactive ammonia uptake by secondary organic aerosol in a changing climate: A WRF-CMAQ evaluation. Front. Environ. Sci 2022, 10, 310.

[55] Zhang R; Thompson TM; Barna MG; Hand JL; McMurray JA; Bell MD; Malm WC; Schichtel BA Source regions contributing to excess reactive nitrogen deposition in the Greater Yellowstone Area (GYA) of the United States. Atmos. Chem. Phys 2018, 18, 12991–13011.

[56] Allen EB; Rao LE; Steers RJ; Bytnerowicz A; Fenn ME Impacts of atmospheric nitrogen deposition on vegetation and soils in Joshua Tree National Park. In The Mojave Desert: Ecosystem Processes and Sustainability; Webb RH, Fenstermaker LF, Heaton JS, Hughson DL, McDonald EV, Miller DM, Eds.; University of Nevada Press: Las Vegas, NV, USA, 2009; pp. 78–100.

[57] Inouye R Effects of shrub removal and nitrogen addition on soil moisture in sagebrush steppe. J. Arid. Environ 2006, 65, 604–618.

[58] Balch JK; Bradley BA; D’Antonio CM; Gómez-Dans J Introduced annual grass increases regional fire activity across the arid western USA (1980–2009). Glob. Chang. Biol 2013, 19, 173–183. 23504729 10.1111/gcb.12046

